# The Threat of the Combined Effect of Biotic and Abiotic Stress Factors in Forestry Under a Changing Climate

**DOI:** 10.3389/fpls.2020.601009

**Published:** 2020-11-30

**Authors:** Demissew Tesfaye Teshome, Godfrey Elijah Zharare, Sanushka Naidoo

**Affiliations:** ^1^Department of Biochemistry, Genetics and Microbiology, Forestry and Agricultural Biotechnology Institute (FABI), University of Pretoria, Pretoria, South Africa; ^2^Department of Agriculture, University of Zululand, KwaDlangezwa, South Africa

**Keywords:** stress interaction, tree growth, tree mortality, forest disease, insect pests, economic impact, response, mitigation

## Abstract

Plants encounter several biotic and abiotic stresses, usually in combination. This results in major economic losses in agriculture and forestry every year. Climate change aggravates the adverse effects of combined stresses and increases such losses. Trees suffer even more from the recurrence of biotic and abiotic stress combinations owing to their long lifecycle. Despite the effort to study the damage from individual stress factors, less attention has been given to the effect of the complex interactions between multiple biotic and abiotic stresses. In this review, we assess the importance, impact, and mitigation strategies of climate change driven interactions between biotic and abiotic stresses in forestry. The ecological and economic importance of biotic and abiotic stresses under different combinations is highlighted by their contribution to the decline of the global forest area through their direct and indirect roles in forest loss and to the decline of biodiversity resulting from local extinction of endangered species of trees, emission of biogenic volatile organic compounds, and reduction in the productivity and quality of forest products and services. The abiotic stress factors such as high temperature and drought increase forest disease and insect pest outbreaks, decrease the growth of trees, and cause tree mortality. Reports of massive tree mortality events caused by “hotter droughts” are increasing all over the world, affecting several genera of trees including some of the most important genera in plantation forests, such as Pine, Poplar, and *Eucalyptus*. While the biotic stress factors such as insect pests, pathogens, and parasitic plants have been reported to be associated with many of these mortality events, a considerable number of the reports have not taken into account the contribution of such biotic factors. The available mitigation strategies also tend to undermine the interactive effect under combined stresses. Thus, this discussion centers on mitigation strategies based on research and innovation, which build on models previously used to curb individual stresses.

## Introduction

Biotic and abiotic stress factors cause major economic losses by reducing yield and quality in agriculture and forestry. A global survey on the major food crops indicated that pathogens, insect pests (hereafter pests), and weeds cause average yield losses ranging from 17.2% in potato up to 30.0% in rice (Savary et al., 2019). Similarly, the major abiotic stresses such as temperature extremes, drought, as well as the deficiency and toxicity of plant nutrients cause up to 51–82% annual loss of crop yield in the world (Oshunsanya et al., 2019). Despite the lack of similar comprehensive assessments of losses, there is sufficient evidence indicating that the forestry sector is similarly affected by these biotic and abiotic stresses (Phillips et al., 2009; Hurley et al., 2017; Graziosi et al., 2019; Schuldt et al., 2020). For example, Forest Resources Assessment (FRA-2015) revealed that the major biotic and abiotic stresses affected 141.6 million ha of forest in 75 reporting countries between 2003 and 2012 (van Lierop et al., 2015). Thus, biotic and abiotic stresses can negatively affect the “ecosystem services” of forests (Alcamo et al., 2003) and may contribute to the decline in the global forest area (Keenan et al., 2015).

The global forest area is expected to continue declining despite the recent decrease in the rate of annual forest loss (d’Annunzio et al., 2015; Keenan et al., 2015) and increase in planted forest area (Payn et al., 2015). According to FRA-2015, while the global forest area decreased from 4.12 to 3.99 billion ha from 1990 to 2015 (Keenan et al., 2015), planted forest area increased from 167.5 to 277.9 million ha during the same period (Payn et al., 2015). d’Annunzio et al. (2015) predicted that the global forest area will continue to decline in the current decade, though at a lower rate of loss. However, Song et al. (2018) found an increase in the overall area of global tree cover between 1982 and 2016. Yet, owing to the observed (Song et al., 2018) and predicted regional differences (d’Annunzio et al., 2015), it is expected that there will be areas, where forests will be lost at a very high rate.

The vulnerability of forests to biotic and abiotic stresses is increasing with climate change (Allen et al., 2015; Pureswaran et al., 2018) and global movement of pathogens and pests (Roy et al., 2014). All scenarios of global climate predictions indicate that the observed global change will continue and cause major changes in precipitation and temperature in different parts of the world (IPCC, 2018). Significant increase in temperature was observed on 76% of the global land area in the 20th century, and a further increase of 2.4–4°C is predicted to occur by 2100 (Gonzalez et al., 2010). Increasing frequency and intensity of droughts accompanied by global warming driven higher temperature, termed “hotter droughts” (Allen et al., 2015), are further witnesses of a changing climate (Crockett and Westerling, 2018). Global land area affected by prolonged heat waves increased from an average of less than 1% in the period from 1951 to 1980 to 10% in the period afterward, reaching as high as 22.21% in 2010 (Hansen et al., 2012) and is expected to increase throughout the 21st century (Wu et al., 2020). Such concerning changes are matched by the threat of forest pathogens and pests, which also continue to show increasing trends in different parts of the world (Roy et al., 2014; Deidda et al., 2016; Hurley et al., 2016; Nahrung and Carnegie, 2020).

Trees are often exposed to both simultaneous and sequential combinations of several biotic and abiotic stresses recurring throughout their long life. Thus, it is important to understand the complex interactions between multiple biotic and abiotic stresses (Anderegg et al., 2015) as it is difficult to predict the response of trees to multiple stress factors and the resulting damage from single stress studies (Pandey et al., 2015). This is particularly urgent in the context of global climate change, which may further complicate the interactions through increasing the frequency and severity of extreme weather events (IPCC, 2018). These events may increase the susceptibility of trees (Buotte et al., 2017), facilitate the spread, reproduction, and development of pathogens and pests (Matsuhashi et al., 2020), and weaken or destroy their natural enemies and competitors (Thurman et al., 2017). While climate change may also reduce damage by negatively affecting pests and pathogens (Zhan et al., 2018), more increased than decreased effects on tree growth and mortality have been observed (Creeden et al., 2014; Camarero et al., 2018).

Despite earlier focus on the dynamics and management of individual stresses in forest trees, research on combined biotic and abiotic stresses is increasing. Recent reviews focused on the mechanistic and theoretical foundations of some interactions (Cobb and Metz, 2017; Jactel et al., 2019; Simler-Williamson et al., 2019) estimated/predicted the effect of some of the interactions (Jactel et al., 2012; Gely et al., 2020), and documented regional impacts (Kolb et al., 2016). While a lot of studies focused on experimental stresses (Desprez-Loustau et al., 2006) and increased our understanding of the physiological and molecular mechanisms of tree responses, the more complex interactions in the field may have different outcomes (Huber and Bauerle, 2016). Most of the previous work focused on the impact of the gross “global change” which includes slight changes in temperature and moisture (Ayres and Lombardero, 2000; Weed et al., 2013; Pureswaran et al., 2018) and may affect the dynamics of forest pathogens and pests without necessarily causing physiological abiotic stresses on trees.

In this review, we discuss the importance and impact of climate change driven interactions between biotic and abiotic stresses in forestry. The damage biotic and abiotic stresses cause to trees could be the best indicator of impact (Jactel et al., 2012) because it is often difficult to partition the effect of individual stresses and their interactions under their combined occurrence (Calvão et al., 2019). Thus, using recent observations from forests in different parts of the world, we show how these interactions will shape the damage from forest disease and pest outbreaks, their effect on tree growth and mortality, as well as the resulting ecological and economic impacts. In addition to the effect of environmental factors under climate change such as variations in precipitation and temperature, we assess how these environmental factors at the level of an abiotic tree stressor will interact with the biotic stress factors and affect trees. We also discuss how the available mitigation strategies can be employed in this context. Despite the importance of the abiotic stresses such as nutrient toxicity and deficiencies, soil salinity and acidity, radiation extremes as well as the biotic stresses such as parasitic plants and mammalian herbivory, we limit our observations to the interactions of pests and pathogens with heat and drought stresses. These combinations represent the most important biotic-abiotic stress interactions inflicting the most damage (Breshears et al., 2005; Carnicer et al., 2011; Fettig et al., 2019; Gheitury et al., 2020), and their impact is increasing as they are strongly affected by global climate change (McDowell et al., 2018). As a result, most of the studies also focus on these interactions (Desprez-Loustau et al., 2006). Furthermore, from the perspective of the physiological and molecular responses of plants, these interactions are generally representatives of many of the biotic-abiotic stress interactions (Wang et al., 2003; Kissoudis et al., 2014).

## Weather Extremes and Forest Disease/Pest Outbreaks

Forest diseases and pests are significant threats to the forest sector. For example, diseases and pests respectively affected at least 12.5 and 85.5 million ha of forest in 75 countries reporting to FRA-2015 between 2003 and 2012 (van Lierop et al., 2015). They were found to be the most important causes of forest disturbance in the Northern Hemisphere affecting 43.9 million ha of forests every year (Kautz et al., 2017). Similarly, a recent study indicated that both pathogens and pests are among the major agents of disturbance in the temperate forests (Sommerfeld et al., 2018).

The interaction among plants, pathogens and pests, and the environment has been an important aspect of plant disease and pest outbreaks. With the increasing frequency and intensity of weather extremes due to global climate change, however, the environment is not just a matter of more or less “optimum” condition for biotic stress factors, rather it also comprises abiotic stress factors which affect the plants directly and indirectly. Similarly, extreme weather events may also affect pathogens and pests, further complicating the interaction. Apart from the weather extremes such as droughts and heat waves, mild variations in temperature and precipitation also affect the dynamics of disease and pest outbreaks (Ayres and Lombardero, 2000; Dukes et al., 2009; Weed et al., 2013).

The impacts of global climate change on forest pathogen and pest populations as well as the mechanisms and theoretical models behind their interaction with weather extremes have been studied using both experimental stresses and field observations (reviewed in Desprez-Loustau et al., 2006; Dukes et al., 2009; Cobb and Metz, 2017; Jactel et al., 2019; Simler-Williamson et al., 2019; Gely et al., 2020). Generally, changes in weather may affect both the host and the pathogen/pest either negatively or positively resulting in either an increase or a decrease in disease/pest outbreaks as well as the subsequent impacts on tree growth and mortality. Nevertheless, mechanistic models focusing on the pathogens and pests themselves indicate the possibilities of increased outbreak as the more likely scenario (Pureswaran et al., 2018; Jactel et al., 2019). It was also previously shown that most of the experimental drought-pathogen infection trials confirmed synergistic interaction (Desprez-Loustau et al., 2006). Similarly, a meta-analysis by Jactel et al. (2012) revealed an overall significant positive effect of drought on pathogen and pest damage. Thus, more rather than less damage can be expected from most of the biotic and abiotic stress combinations under climate change.

Furthermore, the change in the distribution and range of pathogens and pests due to climate change (Burgess et al., 2017; Pureswaran et al., 2018) may increase outbreaks in wider areas. Observed trends show that the area affected by diseases and pests increased from boreal to subtropical forests (Kautz et al., 2017). However, predictions show that climate warming may reverse this trend for some of the important pests and pathogens (Burgess et al., 2017). These changes may ultimately increase the area affected by outbreaks and the resulting damage at a global scale. In this section, we summarize the recent observed and predicted trends in forest disease and pest outbreaks using the damage to trees, except tree growth and mortality, which are discussed in the next sections, as an indicator to the outcome of the complex interactions between pathogens/pests and abiotic stresses (Jactel et al., 2012).

### Forest Disease Outbreak

Variations in temperature and precipitation are observed to affect the prevalence and incidence of forest diseases and may lead to an outbreak (Supplementary Table 1). Several reports indicated that warming temperature increased the prevalence of diseases (Fabre et al., 2011; Brodde et al., 2019; Calvão et al., 2019). However, the effect of precipitation was less consistent showing negative (Calvão et al., 2019), positive (Fabre et al., 2011; Woods et al., 2016; Gao et al., 2019), and no correlation (Brodde et al., 2019; Thoma et al., 2019) with disease. This indicates that areas where an increase in temperature is predicted along with both decrease and increase in precipitation may possibly be exposed to future disease outbreaks. Indeed, Fabre et al. (2011), Bosso et al. (2017), and Matsuhashi et al. (2020) predicted future increases in disease outbreaks under these different scenarios (Supplementary Table 1). Thus, despite the contrasting observations, increased disease outbreaks are likely in many areas as climate predictions are equally contrasting in different regions of the world (IPCC, 2018).

There is also a possibility that changing climate may reduce or does not affect disease outbreak. A notable example for this is that a warming condition is found to be the major driver leading to local extinction of the fungal pathogen *Triphragmium ulmariae* (Zhan et al., 2018). Paap et al. (2017) reported that the incidence of *Phytophtora* spp. on *Corymbia calophylla* did not increase with temperature and only slightly increased with decreasing precipitation. Temperature increased infection of *Pinus albicaulis* by *Cronartium ribicola* only at high relative humidity and up to a certain threshold of 11°C (Thoma et al., 2019). Both extremely low and high temperatures did not favor crown rot disease caused by *Phytophthora alni* in alders (Aguayo et al., 2014) and pine wilt disease caused by pine wood nematode, *Bursaphelenchus xylophilus* (Gao et al., 2019) indicating that warming conditions may well reduce damage in some areas. However, a possible shift in the range of pathogens may cancel out this effect at a regional and global scale. For example, Burgess et al. (2017) predicted that global warming will increase the distribution of *Phytophthora cinnamomi* in cold areas, where it currently does not occur and decrease in warm areas, where it currently occurs. Moreover, observations of reduced impacts are rather scarce in the literature.

The changes in disease outbreaks observed or predicted under different scenarios of moderate and gradual changes in temperature and precipitation regimes may differ under extreme conditions, such as heat and drought stresses. For example, Calvão et al. (2019) demonstrated that the mortality of *Pinus pinaster* under *B. xylophilus* infection is worsened by hotter and drier conditions indicating that these conditions may have enhanced infection. Previously, a review on the interaction of mainly experimental drought with different pathogens in trees revealed that drought and pathogen infection showed synergistic interaction in most of the cases (Desprez-Loustau et al., 2006). However, observations on the effect of heat waves, droughts, and hotter droughts on the incidence and severity of diseases seem to be scarce in the literature. Most of the previous studies focused on the resulting mortality, which will be discussed later.

The effect of drought and heat stresses may vary with the type of disease, the affected tissue, and the level of the abiotic stress (Desprez-Loustau et al., 2006; Jactel et al., 2012). Drought has been shown to significantly increase the damage caused by leaf pathogens and reduce that of root and stem pathogens (Jactel et al., 2012). Recent experimental studies have shown that drought increases the severity of diseases caused by necrotrophic pathogens in Pine and *Eucalyptus* (Sherwood et al., 2015; Barradas et al., 2018). Similarly, the resistance of *Eucalyptus marginata* clones to *P. cinnamomi* decreases with increasing temperature (Hüberli et al., 2002). However, we did not find other observations that support these results.

In general, the available information on the observed and predicted interactions between forest pathogens and changes in temperature and precipitation tend to show more damage than less. In cases where climate change reduces vulnerability to diseases, it is limited to specific pathogens and localities. Furthermore, such effects may be offset by possible new outbreaks related to range shift that can be enhanced by increased distribution of the pathogens due to globalization. This may result in a transitional period of a novel outbreak, which will be exotic to the trees, and is more damaging as has been seen in ash dieback (Marcais et al., 2017). Moreover, we only have limited knowledge on the interactions with the weather extremes such as drought and heat waves, which are predicted to increase in frequency and intensity.

### Insect Pest Outbreak

Temperature is one of the most important drivers of forest pest outbreaks as shown in Supplementary Table 1. Rising temperatures have been associated with increased spruce budworm (*Choristoneura* spp.) outbreaks in North America (De Grandpré et al., 2019) and also increased infestation of *Picea abies* by *Ips typographus* in Europe (Marini et al., 2017; Mezei et al., 2017). This is consistent with the effect of warmer temperature in increasing the reproduction and survival of insects (Pureswaran et al., 2018). However, warming may not affect pest outbreak unless it is synchronized with the important phases of the insect’s life cycle such as overwintering. In this regard, Gazol et al. (2019) showed that only warmer winters affect pine processionary moth, *Thaumetopoea pityocampa*, outbreak. According to these findings, while warming temperature may increase pest outbreaks, there are also possibilities that this may not always be the case.

While moderate warming has been associated with increased pest outbreak, extreme high temperature may have a different effect. For example, the enhancement of *I. typographus* infestation by warming declined at temperatures higher than a certain threshold (Mezei et al., 2017). It has been shown that extremely high temperatures such as heat waves may be lethal to the insects (Rouault et al., 2006). Thus, temperature which is not too high to kill the trees may reduce pest outbreak. Due to limited information on this aspect, there is a need for research to evaluate the temperature thresholds of the important pest species.

Drought has been strongly associated with historic pest outbreaks (Klein et al., 2019). However, its effect varies with the feeding guilds of insects, the substrate they feed on, and the intensity of drought (Jactel et al., 2012; Kolb et al., 2016; Supplementary Table 1). The outbreaks of bark beetles, wood borers, and sap suckers are often associated with drought. For example, drought increased bark beetle outbreaks in the United States (Kolb et al., 2016) and *Sirex noctilio* outbreak in Argentina (Lantschner et al., 2019). However, Jactel et al. (2012) also indicated that drought has a negative effect on sap suckers and wood borers. Yet, massive tree mortality events associated with the combined effect of droughts, and these groups of pests are increasing (see section Tree Mortality).

The effects of drought on defoliator pest outbreaks are not consistent in the literature. Drought decreased outbreaks of larch casebearer, *Coleophora laricella*, in the United States (Ward and Aukema, 2019). Similarly, insect and fungal pathogen caused defoliation decreased during severe drought in Southern European forests as defoliation and tree mortality caused by drought increased (Carnicer et al., 2011). The effect of drought on Western spruce budworm, *Choristoneura freeman*, outbreak varied during the different phases of the outbreak and had contrasting effect on drier and wetter areas. Although drought was a strong driver of outbreak initiation in wetter areas, it had no effect on the expansion of the area affected by the outbreak. In the drier areas, drought had no effect on outbreak initiation and only a small effect on the outbreak expansion (Xu et al., 2019). This indicates that drought stress may have a more important role in the initiation of pest outbreaks than their expansion. While the area affected by and intensity of pine caterpillar, *Dendrolimus* spp., outbreak clearly increased during drought years, the outbreak decreased with increasing precipitation (Bao et al., 2019). Overall, these results indicate that the effect of drought on the outbreak of defoliator insects may be weak, at least at the expansion phase.

From the foregoing discussion, it can be inferred that both drought and warming are associated with an increase in pest outbreak in many cases. However, their effects are not independent of each other. For example, the effect of temperature was observed to decrease with low rainfall (Marini et al., 2017). Several observations indicate that warmer and drier conditions favor outbreaks of many important bark beetles. Hotter droughts increased outbreaks of the mountain pine beetle *Dendroctonus ponderosae* in Western United States (Creeden et al., 2014; Buotte et al., 2017), the Eurasian spruce bark beetle *I. typographus* in Italy (Marini et al., 2012) and Austria (Netherer et al., 2019), and the eastern larch beetle *Dendroctonus simplex* in United States (Ward and Aukema, 2019). Predictions also show that the trend of increased *D. ponderosae* outbreak will continue with increasing temperature and drought in Western United States until 2100 (Buotte et al., 2017). In line with these trends, such combinations have been important drivers of massive tree mortality.

Recent evidence revealed that Norway spruce stands in relatively wetter areas are more susceptible to bark beetle attack during drought seasons than stands in drier areas (Netherer et al., 2019) suggesting a possible acclimation by mild long term moisture deficit. A similar effect was observed in the initiation of Western spruce budworm outbreak (Xu et al., 2019). If this effect is further substantiated, it may give a choice to forest managers between minimizing risk from possible outbreaks and accepting some possible loss in productivity due to moisture deficit during normal times (Lévesque et al., 2014). Thus, further studies are needed on the extent of acclimation and the balance between predisposition and acclimation in different tree species (Bostock et al., 2014).

## The Effect of Combined Biotic and Abiotic Stresses on the Growth and Mortality of Forest Trees

### Tree Growth

Although warmer weather may increase plant growth in wetter areas and currently colder areas such as boreal forests (Torzhkov et al., 2019), weather extremes such as drought and excessively high temperature are among the main tree growth limiting factors (Pichler and Oberhuber, 2007; Lévesque et al., 2014). Plant growth and reproduction are usually negatively correlated with these abiotic stresses (Blum, 2005). Similarly, in defense-growth trade-off, plants reduce growth and reproduction and allocate more resources in defending themselves against pathogens and pests (Jacquet et al., 2012; Huot et al., 2014). Plants may use different mechanisms to regulate this trade-off, which are also dependent on environmental factors (Kliebenstein, 2016; Karasov et al., 2017). Consequently, it is imperative to understand how combined biotic and abiotic stresses will shape this trade-off and affect the growth of forest trees.

The outcome of the interaction between different biotic and abiotic stresses with respect to tree growth varies with the type and level of the stresses and the species of trees. For example, the reduction in the growth of *P. abies* infected by the fungal pathogen *Heterobasidion annosum* was shown to be higher in drier and warmer locations (Gori et al., 2013) showing a synergistic interaction between the two stresses. Given that *H. annosum* is a root pathogen which may affect the transport of water, it can be hypothesized that pathogens which affect the vascular system of trees such as root rot and canker causing pathogens may have a similar effect. Infection by the fungal pathogen *Gremmeniella abietina* has been shown to reduce basal area increment by 26–58% in *Pinus sylvestris* stands (Sikström et al., 2011). Future studies may focus on determining whether drought will further reduce growth in such cases.

Defoliation by pests during drought has been thought to reduce evapotranspiration and hence water deficit stress, thus avoiding damage due to the interaction between pests and water deficit stress (Bouzidi et al., 2019). In support of this, the interaction between climatic moisture index and defoliation by pests enhanced the growth of surviving *Populus tremuloides* despite the negative effect on their survival (Cortini and Comeau, 2020). Even though combined defoliation by pine processionary moth, *T. pityocampa*, and mild drought reduced growth in *P. sylvestris*, this was matched by a similar level of recovery during non-drought years causing no overall loss in growth in the long term (Linares et al., 2014). Growth showed either a weak positive correlation or no correlation with the interaction between pest defoliation and mild drought in non-host and host trees, respectively (Itter et al., 2019). According to these observations, the risks of reduction in tree growth due to combined drought and pest defoliation might be minimal during relatively short term and mild droughts. Conversely, considerable damage due to insect defoliation in areas, where relatively longer and more intense droughts are predicted cannot be ruled out (Balducci et al., 2020).

The effect of either of drought and heat or pathogens and pests on the reduction of growth may be higher depending on the intensity of each of the stresses. The contribution of drought to growth decline was higher than that of insect outbreak in *P. tremuloides* (Chen et al., 2018). However, it was not indicated if there were any interactive effects. While leaf damage by the aspen leaf miner, *Phyllocnistis populiella*, reduced basal area index, variation in climatic moisture index and its interaction with defoliation had no effect on growth (Boyd et al., 2019). Nonetheless, the interaction of pest defoliation with severe drought may have a different impact on tree growth. For example, the growth of *P. tremuloides* decreased by 33% under the combined effect of hotter drought, defoliator, and wood boring pests between 1997 and 2014 in Canada (Hogg et al., 2008). These results indicate that the interactions of more severe abiotic stresses with pests and pathogens may cause more severe damage to tree growth.

### Tree Mortality

Tree mortality is a natural phenomenon often caused by several contributing factors. Generally, it occurs at a very low rate in all forest populations without causing any considerable ecological and economic damage in the short term (Jimenez et al., 1985). Tree mortality events which affect relatively larger areas and a large number of trees have been mainly associated with rare catastrophic events, such as hurricanes, earthquakes, and landslides (Jimenez et al., 1985; Lugo and Scatena, 1996). However, recent evidences show that large scale tree mortality events, which are not associated with such rare catastrophes are increasing (Allen et al., 2010, 2015; Greenwood et al., 2017; McDowell et al., 2018) Here, we use the tree mortality classification given in Lugo and Scatena (1996) with the exception that the term “massive mortality” will be used to represent the mortality events, which can be considered as both “catastrophic” and “extensive/massive.”

The abiotic stresses such as the extremes of moisture and temperature as well as the biotic stresses such as pests and pathogens have been among the major drivers of massive mortality events (McDowell et al., 2018). It has long been known that tree mortality is a result of the contribution of several interacting causes that involve both biotic and abiotic factors (Franklin et al., 1987; Ciesla and Donaubauer, 1994). Thus, it can be expected that the different biotic and abiotic stresses are more likely to be acting in combination to cause the increasing massive mortality events. Indeed, there is an increasing number of observations in support of this (De Grandpré et al., 2019; Stephenson et al., 2019; Ward and Aukema, 2019). Even though these stress factors have always been problems in forestry and agriculture, the intensity and frequency of extreme weather events as well as forest disease and pest outbreaks have increased and will continue to increase due to climate change and global movement of pests and pathogens (Hansen et al., 2012; Roy et al., 2014; IPCC, 2018).

**Figure 1:**
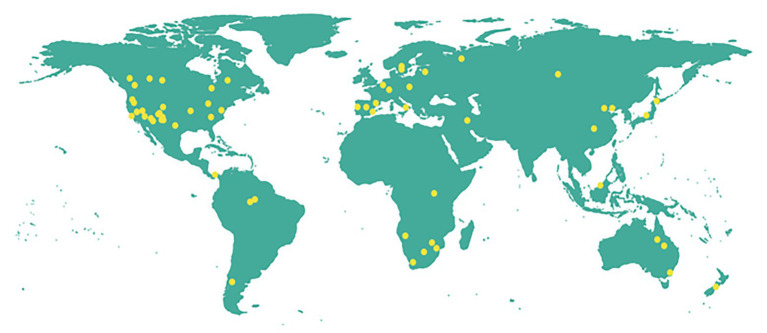
Map showing recent (1982–2020) landscape level tree mortality events associated with the biotic and abiotic stresses drought, heat, pests, pathogens, and their combinations reported in peer reviewed publications. The summary of the reports and the respective references are given in Supplementary Table 2. We searched on Google scholar using different combinations of the key words: “tree,” “forest,” “vegetation,” “plantation,” “massive,” “mortality,” “die-off,” “die-back,” and “decline.” Reports from relevant reviews (Allen et al., 2010, 2015; McDowell et al., 2018) were also searched. Reports which do not indicate the spatial characteristics and intensity of mortality as well as those which include other exogenous mortality agents such as fire, flood, etc., were excluded. In addition, seedling mortality, mortality due to experimental stress, long term mortality analyses were not included. Then, reports which clearly describe individual mortality event/events and can be approximated as landscape scale according to the classification given in Lugo and Scatena (1996) were selected. Reports describing different aspects of the same mortality event at the same location were considered as single reports.

**Figure 2:**
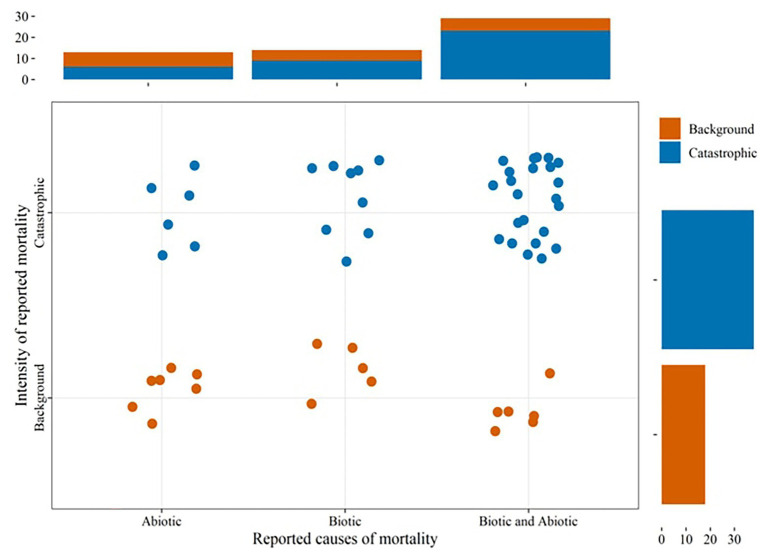
The proportion of landscape level tree mortality events (Figure 1; Supplementary Table 2) reporting single or combined biotic and abiotic stresses as causes. The intensity of mortality events were approximated to background and catastrophic based on the classification given in Lugo and Scatena (1996).

Reports of landscape level tree mortality events which can be considered to occur at both background and catastrophic rates (Lugo and Scatena, 1996) are increasing in different parts of the world (Figure 1; Supplementary Table 2). Combined biotic and abiotic stresses have been associated with many of these events, most of which are massive mortality events (Figure 2). This underlines that recent massive mortality events are more likely driven by combined biotic and abiotic stresses. This is in line with the “coupling” among various drivers and mechanisms of tree mortality hypothesized in McDowell et al. (2018), where drivers of tree mortality such as drought, high temperature, and biotic factors interact through the physiological mechanisms of tree death such as carbon starvation and hydraulic failure.

**Figure 3:**
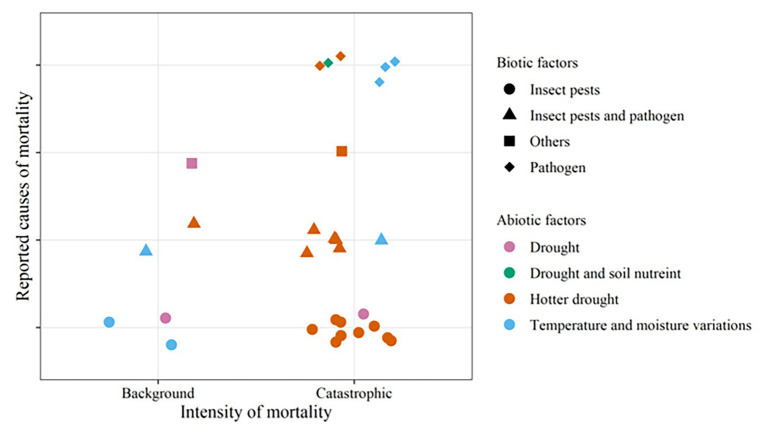
The main biotic-abiotic stress combinations associated with recent landscape level tree mortality events caused by combined stress (Figure 2; Supplementary Table 2). Normal droughts are differentiated from hotter droughts, which refer to droughts accompanied by increased temperature as termed in Allen et al. (2015). “Others” refers to parasitic plants alone or with insect pests. The symbols/plot characters represent mortality events caused by combined stress. The shape and colors of the characters respectively represent the biotic and abiotic factors involved.

Hotter droughts have been associated with most of the massive mortality events, and both pathogens and pests have been reported in most of them (Figure 3). Some of the most severe recent mortality events are results of the combined effects of hotter droughts, bark beetles, and fungal pathogens (Worrall et al., 2008, 2010; Klockow et al., 2018; Gheitury et al., 2020). The combination between hotter droughts and bark beetles (Breshears et al., 2005; Floyd et al., 2009; Millar et al., 2012; de La Serrana et al., 2015; Kharuk et al., 2019) as well as hotter droughts and pathogens (Holuša et al., 2018; Wood et al., 2018) have also resulted in severe losses. These are considerable threats to forestry in the future as predictions show that hotter droughts (Crockett and Westerling, 2018) and associated tree mortality (Zhang et al., 2014b; Hember et al., 2017) have been increasing in different parts of the world and are expected to continue to increase (Allen et al., 2015; Wu et al., 2020).

Previously, Ciesla and Donaubauer (1994) established that abiotic stresses act as predisposing factors to biotic attacks which usually come out to be the inciting and contributing factors to tree death. A recent example for this was reported in Ward and Aukema (2019), where changes in temperature and precipitation predisposed eastern larch, *Larix laricina*, to defoliation by larch casebearer, *C. laricella*, and finally tree mortality occurs after eastern larch beetle, *D. simplex*, infestation. Furthermore, delayed mortality associated with pathogens and pests was observed after a severe drought indicating a possible predisposition by drought (Klockow et al., 2018). Thus, mortality events seemingly caused by a single biotic or abiotic stress may well have unreported biotic or abiotic predisposing/inciting factors. However, in some of the landscape level tree mortality events caused by droughts, biotic stress factors were not involved (Figure 3). Similarly, Kautz et al. (2017) estimated that biotic disturbances may cause up to 3.3 million ha tree mortality in the Northern Hemisphere per year without the involvement of abiotic stress factors. Re-examination of such mortality events will provide an insight in this regard. Future studies and prediction models should therefore consider both biotic and abiotic factors.

Parasitic plants and soil nutrients were reported to be associated with a few of the massive mortality events (Figure 3). Warming climate with lower precipitation increased tree mortality due to parasitic plants through decreasing the growth of trees and predisposing them to other drivers of mortality (Galiano et al., 2010; Bell et al., 2019). Increased infestation with insects and parasitic plants after severe drought was associated with a large increase in the annual tree mortality in pinyon pine (Flake and Weisberg, 2019). Soil nutrients, drought, and bacterial canker were found to cause massive poplar mortality in China (Ji et al., 2019). Thus, although these factors are beyond the scope of this review, they deserve to be explored in future research.

**Figure 4:**
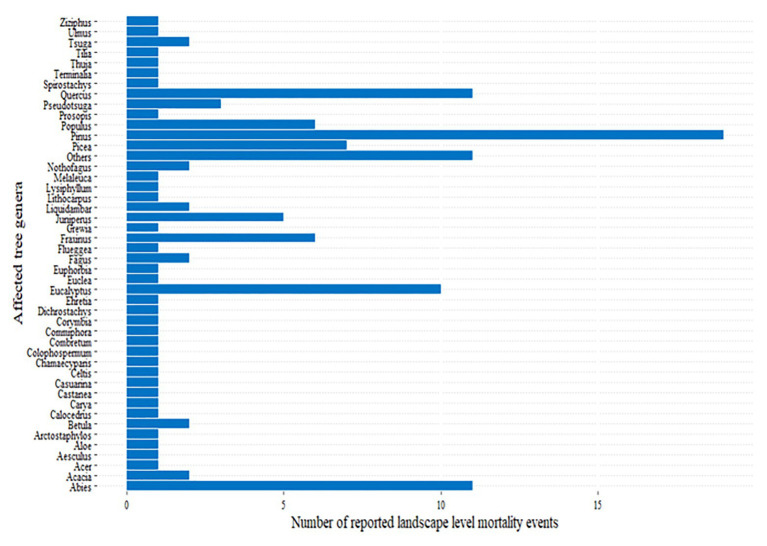
The main genera of trees affected by landscape level tree mortality events across the world (Figure 1; Supplementary Table 2).

The reported landscape level mortality events (Figure 1) affected different species of trees belonging to several genera (Figure 4), including some of the most planted genera, such as *Pinus*, *Eucalyptus*, *Populus*, *Picea*, and *Abies* (Del Lungo et al., 2003; Brockerhoff et al., 2008). The genus *Pinus*, which covers the largest area of planted forests in the world (Del Lungo et al., 2003), is also the most highly affected genera (Figure 4). For example, mortality due to the combined effect of hotter drought and *Ips confuses* infestation in the United States was estimated to reach as high as 80% in *Pinus edulis* affecting more than 1.2 million ha forest during 2000–2003 (Breshears et al., 2005). Similarly, 89.6 and 48.1% of sampled *Pinus ponderosa* and *Pinus lambertiana* trees, respectively, were dead under the combined effect of *bark beetles* and the 2012–2015 hotter drought in California (Fettig et al., 2019). These mortality events clearly affected some species more severely than others (Suarez et al., 2004; Floyd et al., 2009; Fettig et al., 2019). Thus, forest tree genetic improvement programs and forest managers should consider selecting tree species that are tolerant to these combined stresses.

The effect of combined biotic and abiotic stresses on tree mortality is not limited to the extensive and catastrophic events on which we mainly focused here. There is evidence showing that the rate of gradual background tree mortality is also increasing with changing climate (van Mantgem et al., 2009; Taccoen et al., 2019). The biotic stress factors such as pests and pathogens have been identified as the major drivers of background tree mortality (Das et al., 2016). Therefore, it is reasonable to hypothesize that non-outbreak levels of pathogens and pests interacting with mild climate change driven abiotic stresses may be responsible for the increasing rate of background tree mortality. Recent evidence in support of this is that temperature and moisture variations indirectly affected pest and pathogen driven deaths of *Abies lasiocarpa* (Lalande et al., 2020).

## Economic and Ecological Impacts of Combined Biotic and Abiotic Stresses in Forestry

The major causes of forest loss leading to the decline of the global forest area remain to be deforestation, shifting cultivation and wildfire (Curtis et al., 2018). While the direct contribution of biotic and abiotic stresses to such forest losses seems to be relatively low (Curtis et al., 2018), their indirect role cannot be underestimated. For example, tree mortality caused by insect pest outbreaks, heat waves, and droughts are frequently associated with forest fires resulting in huge tree losses (Brando et al., 2014; Klein et al., 2019; Talucci and Krawchuk, 2019; Xie et al., 2020). In addition, the regional and temporal variation in the occurrence of biotic and abiotic stresses also highlights the importance of these factors. For example, pests were reported to cause 32% of the tree mortality in the Western United States compared with 18% loss caused by fire (Berner et al., 2017). Another study also indicated that biotic disturbances such as pathogens and pests are the most important causes of forest disturbance in the forests of the Northern Hemisphere (Kautz et al., 2017).

Apart from complete forest loss that leads to a change in land use, the economic and ecological impact of biotic and abiotic stresses operating in forestry can be viewed through their impact on the ecosystem services of trees (Alcamo et al., 2003). Previously, Boyd et al. (2013) used this framework to summarize the impact of pests and pathogens. The decline in forest productivity due to tree mortality and reduced growth resulting from combined biotic and abiotic stresses (section The Effect of Combined Biotic and Abiotic Stresses on the Growth and Mortality of Forest Trees) as well as reduced quality of products (Brodde et al., 2019) can affect the provisioning services and cause a direct revenue loss (Zwolinski et al., 1990; Aukema et al., 2011). Though controversial, it has been argued that climate change increases tree growth and hence forest productivity (Kirilenko and Sedjo, 2007; Reyer et al., 2017; Torzhkov et al., 2019; Ruiz-Pérez and Vico, 2020). However, even if there would be a possible increase, the impact of extreme weather interacting with increased pathogen and pest outbreaks will cause major losses and may even offset any gain in productivity (Reyer et al., 2017; Woods and Watts, 2019).

Increased tree mortality, crown die-back and defoliation caused by combined biotic and abiotic stresses may have a negative impact on human well-being by affecting the cultural and regulatory services of trees (Alcamo et al., 2003). The decrease in the density of forests and canopy cover of trees have been associated with increased human health problems stemming from respiratory diseases (Donovan et al., 2013) as well as increased temperature associated with the loss of canopy shade (Jones, 2019). Massive tree mortality may also have an impact on other components of the forest ecosystem, such as the micro and macro faunal and floral diversity. For example, massive ash (*Fraxinus* spp.) mortality caused by emerald ash borer (*Agrilus planipennis*) created canopy gap (Ulyshen et al., 2011) and accumulation of woody debris (Perry and Herms, 2017), which affect the activity and diversity of forest invertebrates. Massive tree mortality may also cause a decline in the population of coexisting organisms such as lichens, and may lead to local extinction (Jönsson and Thor, 2012; Lõhmus and Runnel, 2014).

Recent evidence indicates that biotic and abiotic stresses may contribute to the decline in the population of tree species and may even lead to extinction. A good example for this is the fungal pathogen *Austropuccinia psidii*, which has caused a rapid decline in *Rhodomyrtus psidioides* population in Australia since 2012 (Fensham et al., 2020). The coupling of biotic stresses with weather extremes may be beyond the capability of some species to adapt to a changing climate (Schaberg et al., 2008; Sáenz-Romero et al., 2020). This may result in a selective massive death of certain vulnerable species (Suarez et al., 2004) and may lead to local extirpations (Alfaro et al., 2014) and even extinction in the case of endemic species (Fensham et al., 2020). Thus, if the episodes of massive tree mortality caused by combined biotic and abiotic stresses (section The Effect of Combined Biotic and Abiotic Stresses on the Growth and Mortality of Forest Trees) continue even at current pace, the direct and indirect contribution of such stresses to the extinction of tree species may become a real threat at least to the already endangered species.

Biotic and abiotic stresses induce considerable emission of biogenic volatile organic compounds associated with the responses of stressed living trees (Faiola and Taipale, 2020) and decay of dead trees (Kurz et al., 2008; Phillips et al., 2009). Pest attack increased biogenic emissions of different compounds from trees and simultaneously occurring abiotic stresses such as heat and drought mostly further increased such emissions (Faiola and Taipale, 2020). Biotic and abiotic stresses negatively affect the global carbon pool by the loss of potential carbon sinks through reduced growth and death of trees as well as the addition of carbon sources for future emission from decaying dead trees (Kurz et al., 2008; Phillips et al., 2009). For example, hotter drought in the Amazon forest in 2005 caused the loss of 1.21–1.60 Pg potential carbon storage from reduced growth and tree mortality (Phillips et al., 2009). A severe drought in Texas, United States, caused the loss of 24–30 Tg C due to tree mortality (Moore et al., 2016). Similarly, a study in Canada estimated the loss of carbon storage due to pests to be 2.87 tone C ha^−1^ year^−1^ (Zhang et al., 2014a). Furthermore, predictions also indicate that increased drought and associated pest outbreak will significantly affect the carbon balance in a similar fashion (Scheller et al., 2018). These examples are good indicators of the significance of combined biotic and abiotic stresses to environmental sustainability. However, in most of the cases, attempts to quantify these impacts are inadequate. Thus, further research on quantifying the emissions and their environmental impact will benefit environmental models for carbon balance (Faiola and Taipale, 2020).

It is difficult to attach an economic value to all kinds of damages caused by biotic and abiotic stresses. However, there were attempts to estimate the economic impacts from different perspectives (Supplementary Table 3). The economic loss due to tree death and reduced growth is a direct indicator of such impacts. However, dead trees, especially mature ones, can still be of economic value through “salvage logging” despite the undesirable ecological consequences due to the associated increase in harvest frequency (Thorn et al., 2018). An estimate of economic loss derived from predicted tree mortality (Waring et al., 2009; Ochuodho et al., 2012; Soliman et al., 2012), comparisons of the cost of protection to the possible loss (Watt et al., 2011; Cameron et al., 2018), and revenue loss due to downgraded products (Costanza et al., 2019) were used to demonstrate possible damage from biotic and abiotic stresses. Government and household expenditures as well as losses in property value associated with tree mortality have also been estimated (Aukema et al., 2011). More holistic assessments included the economic loss from production, protection, tourism, and carbon sequestration (Notaro et al., 2009). However, only some of the studies (Waring et al., 2009; Ochuodho et al., 2012; Soliman et al., 2012) considered the combined effects of biotic and abiotic factors, which may result in an over- or under-estimation of loss. Because damages such as tree mortality are mostly the results of the combined effect of biotic and abiotic stresses, future studies should include these factors into their analyses. Moreover, as economic analysis is important for policy makers and forest managers, such information, which may be largely found in technical reports, should be systematically analyzed.

## Responses of Forest Trees to Combined Biotic and Abiotic Stresses

The impact of combined biotic and abiotic stresses on the physiology of trees is different from that of individual stresses. Sequential or simultaneous combinations of biotic and abiotic stresses may have a negative or positive outcome on different morphological and physiological traits of forest trees depending on the species of trees, the type of biotic stress factors, and the duration and intensity of abiotic stresses (Supplementary Table 4). These changes may make trees either more susceptible or resistant to one or more of the co-occurring stresses.

Several individual biotic and abiotic stresses affect plant-water relations. For example, drought stress (McKiernan et al., 2017) and infection by fungal pathogens, which affect the vascular system (da Silva et al., 2018) influence the movement of water and reduce stem and leaf water potential. The simultaneous occurrence of these stresses may cause further reduction in water potential in plants. In support of this, it has been reported that infections by *Neofusicoccum eucalyptorum* in *Eucalyptus globulus* (Barradas et al., 2018) and *Obolarina persica* as well as *Biscogniauxia mediterranea* in *Quercus brantii* (Ghanbary et al., 2017) caused a further reduction in the stem water potential of drought stressed plants. However, this may not always be the case depending on the level of resistance to the involved pathogen as well as the intensity and duration of drought. For example, while both drought and infection by *Quambalaria coyrecup* reduced leaf water potential in *C. calophylla*, their combination did not result in a further reduction (Hossain et al., 2019). Similarly, while water potential decreased due to drought stress, no such reduction was observed due to infection by *Leptographium wingfieldii* in *P. sylvestris* (Croisé et al., 2001) and *P. cinnamomi* in *Quercus ilex* and *Quercus cerris* (Turco et al., 2004). Furthermore, priming with previous drought resulted in significantly higher leaf water potential as compared to non-primed plants under combined drought and *N. eucalyptorum* infection in *E. globulus* (Barradas et al., 2018). According to these observations, while drought stress strongly influences plant water-relations, the effect of fungal pathogens both as an individual stress and in combination with short term experimental drought seems to be moderate.

It is well-known that drought stress negatively affects photosynthetic gas exchange, however, some pathogens (da Silva et al., 2018) and their combination with drought (Supplementary Table 4) have also been reported to have a similar impact. Ghanbary et al. (2017) reported that drought and the pathogens *O. persica* and *B. mediterranea* significantly reduced stomatal conductance, photosynthetic rate, and maximum photochemical efficiency of photosystem II (Fv/Fm) in *Q. brantii.* Interestingly, the combination of both pathogens with drought caused further reduction in all of these parameters. Combined biotic and abiotic stresses may also negatively affect photosynthesis by reducing the concentration of photosynthetic pigments. For example, Ghanbary et al. (2018) reported that chlorophyll content decreased due to pathogen infection, drought, and their combination in *Q. brantii*. These findings indicate that the effect of combined biotic and abiotic stresses on photosynthesis may be worse than each of the individual stresses. This may be one of the reasons for the reduction in tree growth and increase in tree mortality associated with combined stresses.

Accumulation of osmolytes and soluble sugars are among the most common responses of plants to osmotic stress resulting from abiotic stresses such as drought. Such accumulations may increase due to pathogens and pests which affect plant-water relations. Sherwood et al. (2015) and Ghanbary et al. (2018) revealed that proline, which increased under both drought and pathogen infection, showed a further increase under the combination of both stresses. On the other hand, the accumulation of osmolytes and soluble sugars in response to abiotic stresses may create favorable condition for biotic stress factors such as fungal pathogens and wood boring pests thereby increasing the susceptibility of trees. In support of this, Caldeira et al. (2002) reported that a reduced bark moisture content and increased accumulation of glucose, fructose, and sucrose enhanced the survival and growth of *Phoracantha semipunctata* under drought conditions in *E. globulus*. Similarly, combined drought and pathogen infection increased soluble sugar concentration in *Q. brantii* (Ghanbary et al., 2018) resulting in increased susceptibility to pathogens (Ghanbary et al., 2017). These findings indicate that osmotic adjustment may represent a synergistic interaction between responses to biotic and abiotic stresses resulting in increased damage under multiple stress situations.

Metabolites such as phenolics and terpenoids, which are commonly involved in plant defense against pathogens and pests, may also be affected by co-occurring abiotic stress factors, such as heat and drought. Total phenol concentration increased under both drought and pathogen infection as individual stresses in *Q. brantii*, and a further increase was observed under their combination (Ghanbary et al., 2018). The interaction between drought and pine weevil, *Hylobius abietis*, attack respectively decreased and increased the accumulation of polyphenols and diterpins in *Pinus halepensis* (Suárez-Vidal et al., 2019). By doing so, moderate drought weakened basal defense and significantly increased the susceptibility of seedlings to pest attack. However, there were also observations which revealed that combined stress did not have a significant effect on phenol and terpenoid concentrations. For example, a study in *C. calophylla* found that total phenols and total terpenes generally tended to increase due to *Q. coyrecup* infection while drought stress generally did not further increase their concentration (Hossain et al., 2019). Similarly, the concentration of total monoterpenes increased due to infection of *Pinus contorta* and *Pinus banksiana* by *Grosmannia clavigera* while drought stress had no effect (Lusebrink et al., 2016). These variations may be related to the tolerance/resistance of trees to each of the stresses.

Formation of reactive oxygen species (ROS) and their subsequent detoxification is a common response of plants to both biotic and abiotic stresses. Reactive oxygen species along with phytohormone signaling pathways have been considered to be two of the main “converging points” between responses to biotic and abiotic stresses in plants (Zhang and Sonnewald, 2017). Infection by *N. eucalyptorum* reduced the accumulation of Malondialdehyde (MDA) in drought primed *E. globulus* plants as compared to simultaneously infected ones (Barradas et al., 2018). However, this does not indicate improved resistance to the pathogen as lesion length was significantly longer in the drought-predisposed plants. Thus, it was hypothesized that the reduction in MDA may be the result of the pathogen’s defense against pre-accumulated ROS. In agreement with this, despite the increase in MDA concentration due to drought stress, pathogen infection, and their combination in *Q. brantii*, both the highest MDA concentration and the largest lesion were recorded under combined stress (Ghanbary et al., 2017, 2018). Although H_2_O_2_, which increased due to drought stress, significantly decreased upon infection by *Diplodia sapinea* alone, and in combination with drought in *Pinus nigra*, this was not associated with an increased resistance to the pathogen (Sherwood et al., 2015). These results may indicate that the effect of combined biotic and abiotic stresses on the ROS signaling pathway tend to be synergistic resulting in increased damage to trees. However, as hypothesized in Sherwood et al. (2015), this may be limited to necrotrophic pathogens as ROS may affect biotrophs differently. The increased accumulation of ROS due to abiotic stresses may enhance hypersensitive response, which is an effective defense strategy against biotrophic pathogens unlike necrotrophic ones (Zhang and Sonnewald, 2017).

The involvement of phytohormones in modulating growth and responses to both biotic and abiotic stresses is well-known. A review by Zhang and Sonnewald (2017) hypothesized that Auxin may coordinate response to combined biotic and abiotic stresses. A study on *Lycopersicon esculentum* revealed that increased abscisic acid concentration due to drought stress did not cause susceptibility to infection by *Oidium neolycopersici* and *Botrytis cinerea* (Achuo et al., 2006). Similarly, the increased accumulation of jasmonic acid and salicylic acid, as well as unchanged concentration of abscisic acid due to prior drought stress resulted in improved resistance to infection by *Pseudomonas syringae* in *Arabidopsis thaliana* (Gupta et al., 2017). However, we did not find similar studies on forest trees.

The molecular response of plants to combined stress is generally different from their response to individual stresses. In addition to the molecular responses which are shared between the individual biotic and abiotic stresses that may be prioritized depending on the severity of stress, plants show molecular responses which are unique to combined stresses (Choudhary et al., 2016). A number of differentially expressed genes which were shared among the individual stresses and unique to combined stress have been identified in *Arabidopsis* (Gupta et al., 2016; Choudhary et al., 2017). Some of the uniquely regulated genes due to combined drought and pathogen infection in *Arabidopsis* include genes involved in fatty acid and amino acid metabolism, secondary metabolites, and photosynthesis pathways, as well as genes in the transcription factor families such as NAC, WRKY, and MYB (Gupta et al., 2016). However, to the best of our knowledge, there has been no study on the molecular changes due to combined biotic and abiotic stresses in forest trees.

Recent studies in *Arabidopsis* reported the identification of key genes which confer resistance to combined biotic and abiotic stresses. The transcription factor gene *G-Box Binding Factor 3* (*GBF3*), which regulates genes in the ABA signaling pathway (Dixit et al., 2019) and the micro-RNA gene *ath-miR164c*, which regulates genes involved in proline biosynthesis (Gupta et al., 2020), were found to confer tolerance to combined drought and infection by *P. syringae* in *A. thaliana*. Future studies should target the identification and characterization of more common regulators, while research in forest trees should prioritize the investigation of these genes.

## Mitigation Strategies

Prevention strategies such as strict quarantine have been useful in minimizing the introduction of exotic pathogens and pests (Wingfield et al., 2015). In the case of combined biotic and abiotic stresses, prevention is still useful as it minimizes parts of the problem. Besides, it remains to be one of the most important ways to protect our natural forests where other strategies such as genetic improvement of tree resistance are not feasible. However, the possible risk of increased outbreaks of domestic pests and diseases due to climate change (section Weather Extremes and Forest Disease/Pest Outbreaks) calls for better coping mechanisms. Thus, *ex situ* conservation and selection of resistant trees should be considered for endangered species of vulnerable natural forests (Fensham et al., 2020; Sáenz-Romero et al., 2020).

Genetic improvement of tree resistance/tolerance to biotic and abiotic stresses through conventional breeding techniques and genetic engineering is a relatively longer term strategy (Wingfield et al., 2015; Naidoo et al., 2019). The vulnerability of trees to growth decline and massive mortality under the combined effect of biotic and abiotic stresses varied among species (Suarez et al., 2004; Floyd et al., 2009). Such genetic diversity is a valuable resource, not only for selective planting, but also for selective breeding and genetic engineering. The increasing availability of large multi-omics data, systems and synthetic biology approaches as well as improved functional testing will allow us to integrate and complement conventional breeding, genetic engineering, and genome editing (Naidoo et al., 2019). Genetic improvement for combined biotic and abiotic stress tolerance may target either the regulators common to the different stresses or pyramiding of genes governing response to individual stresses (Kissoudis et al., 2014). Recent studies are shedding light on the possibilities of engineering plants for multiple traits (Cho et al., 2019). Thus, improving trees for resistance to combined biotic and abiotic stresses using these techniques may also become possible. Existing model systems that have been used to study biotic stresses such as those in *Eucalyptus* (Naidoo et al., 2013; Mangwanda et al., 2015; Visser et al., 2015), Pine (Visser et al., 2018), and Poplar (Feau et al., 2007; Hacquard et al., 2011) can be used to develop a workable approach in this regard. We propose the use of drought-pathogen interactions, which has been considered a model in annual crops (Pandey et al., 2017), to study combined biotic and abiotic stresses in forest trees. Owing to the representativeness of drought in several abiotic stresses (Wang et al., 2003) and the relative ease of manipulating pathogens in both field and green house studies, drought-pathogen interactions is a suitable model to study combined stress. In addition, as much as drought and pathogens are two of the most important stresses in agriculture and forestry, they have been studied as single and combined factors better than several others and their combinations (Desprez-Loustau et al., 2006; Pandey et al., 2017).

While genetic improvement of trees is a valuable strategy for plantation forestry, it is less feasible to natural forests, and it is also a long-term project which needs initial investment in research. Thus, the options of biological control of pests and pathogens (Hurley et al., 2012; Martín-García et al., 2019) and the use of microorganisms such as mycorrhiza and endophytes to alleviate abiotic stress tolerance (Liu et al., 2015; Khan et al., 2016; Ferus et al., 2019) should be explored. However, these biological agents have been used against single stresses under optimum environmental conditions (Slippers et al., 2012), and thus, may not function under multiple stress settings. For example, drought has been reported to negatively affect biological control using entomopathogenic nematodes (Hassani-Kakhki et al., 2019). Similarly, while severe drought increases *S. noctilio* outbreak (Lantschner et al., 2019), drought has the opposite effect on the biocontrol agent *Deladenus siricidicola* (Hurley et al., 2008). Thus, genetic improvement can and should also target biological control agents (Wang and Wang, 2017).

A number of forest management strategies can be deployed to mitigate the impacts of combined biotic and abiotic stresses. They include; thinning and reduction of the basal area of stands (Bradford and Bell, 2017; Restaino et al., 2019; Lalande et al., 2020), facilitating regeneration in advance of predicted hotter droughts (Redmond et al., 2018), shorter rotation age to minimize damage from bark beetle and droughts (Maclauchlan, 2016), and stand diversification such as “clonal composites” (Rezende et al., 2019). Accurate predictions of massive tree mortality and early warning on hot spots of combined biotic and abiotic stresses (Roux et al., 2015; Preisler et al., 2017; Rogers et al., 2018) will aid not only decision making by forest managers but also scientific interventions and priorities for *ex situ* conservation.

## Conclusion

**Figure 5:**
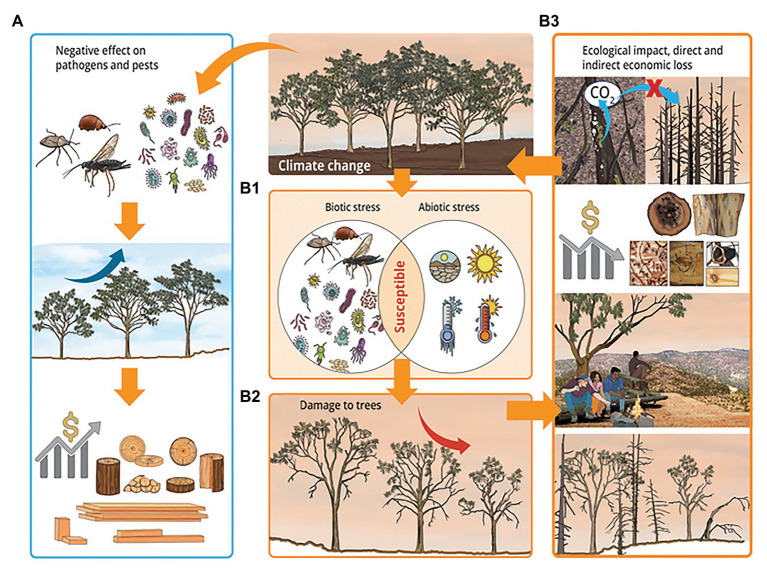
A scheme showing the impact of combined biotic and abiotic stresses in forestry under the influence of climate change. Climate change may reduce damage from diseases and pests by negatively affecting pathogens and insect pests, increase tree growth, and result in beneficial economic and ecological impacts **(A)**. Climate change, along with increased global distribution of pathogens and pests due to globalization, may increase disease and pest outbreaks as well as the intensity and frequency of abiotic stresses. This increases the susceptibly of tress under combined biotic and abiotic stresses **(B1)**, and result in increased damage to trees, which includes decline in tree growth, increase in tree mortality, defoliation, and crown die-back **(B2)**. These damages negatively affect the ecosystem services of trees resulting in harmful economic and ecological impacts **(B3)**. The negative effect on the ecosystem services of forests include loss of potential carbon sinks and increased emission of biogenic volatile organic compounds, reduction in the quality and productivity of forest products, negative impact on human health and well-being, and loss of micro and macro faunal- and floral-diversities, which may in turn cause loss of indirect services from the forest. Yellow arrows indicate cause and effect relationships, and deep blue and red arrows indicate increase and decrease in tree growth, respectively.

Biotic and abiotic stresses have always been important in agriculture and forestry. In recent years, their importance has increased as a result of climate change enhanced frequency and intensity of weather extremes as well as globalization which has increased the movement of pathogens and pests. Plants often face these biotic and abiotic stresses in combination, either simultaneously or sequentially. Forest trees are exposed to the recurrence of these combinations due to their long lifecycle. Plants show both shared and unique responses to combined biotic and abiotic stresses. As a result, it is difficult to predict both the response of plants to and damage due to combined stresses from single stress studies. In this context, we have shown the importance of combined biotic and abiotic stresses as drivers of forest disease and pest outbreaks (Figure 5). Indeed, observed and predicted evidences indicated that combined biotic and abiotic stresses are associated with reductions in tree growth and increasing episodes of massive tree mortality, which have huge economic and ecological implications.

Climate change driven abiotic stresses such as heat and drought may either increase or decrease pest and disease outbreaks depending on the species of trees, pests, pathogens, and forest biomes. Whether the increase or decrease is more likely at a global scale is a subject of continued debate, although the available evidences tend to show more increase in many cases. However, what is more important is that such changes along with the global movement of pathogens and pests will undoubtedly continue to bring in a new spatial and temporal trend of disease and pest outbreaks and the associated damage. This may also couple with weather extremes which are increasing in frequency and intensity.

The current studies and reviews, despite the inconsistency and contradiction of findings, underline two things. First, many of the studies used climatic variables rather than considering the physiological stress caused by weather extremes such as heat and drought. Particularly, while studies on warming showed considerable interaction with pathogens and pests, extreme heat and cold which cause physiological stress to both the trees and pathogens/pests might have an entirely different outcome. The more frequent heat waves and hotter droughts should thus be used as good opportunities to study such responses. Second, the interactions between host trees and pathogens/pests can be affected by climate change driven changes in the abiotic stresses in a complex manner, which can further be complicated by the interacting effect of the different abiotic factors. As a result, despite the global attention given to climate change and its impacts in forestry, we are far from fully understanding the constantly changing conditions. Thus, understanding all levels of interactions at least for the major stress combinations is important. In this regard, both experimental and observational studies using model systems can better equip us to respond to possible damages from combined biotic and abiotic stresses in the future.

## Author Contributions

SN and DT contributed to the conception of the review. DT wrote first draft. SN, DT, and GZ commented and revised the manuscript. All authors contributed to the article and approved the submitted version.

### Conflict of Interest

The authors declare that the research was conducted in the absence of any commercial or financial relationships that could be construed as a potential conflict of interest.
