# Supplementary material for: The Threat of the Combined Effect of Biotic and Abiotic Stress Factors in Forestry Under a Changing Climate
Source: Front Plant Sci. 2020 Nov 30;11:601009. doi: 10.3389/fpls.2020.601009 (PMC7733969; doi:10.3389/fpls.2020.601009)
Supplement: Supplementary file 1 [file Table_1.docx]

Supplementary Material

## Supplementary Table 1. Examples of observed and predicted changes in forest disease and pest outbreaks associated with combined biotic and abiotic stress factors^*^

| **S. No.** | **Insect pest or pathosystem** | **Country/ region** | **Observed/predicted abiotic factor**** | **Observed/predicted trend in disease or insect pest damage level** | **Reference** |
| --- | --- | --- | --- | --- | --- |
| **Diseases, observed** | | | | | |
| 1 | *Diplodia sapinea* on *Pinus sylvestris* | Northern Europe | Temperature | Warmer temperature increased disease levels | (Brodde et al., 2019) |
|  |  |  | Precipitation | No relation |  |
| 2 | *Diplodia pinea* on *Pinus spp*. | France | Temperature and summer rain | Increases in temperature and summer rain increased prevalence | (Fabre et al., 2011) |
| 3 | *Phytophthora alni* on alders (*Alnus spp.*) | France | Temperature | Both low and high temperature in in winter and summer respectively were negatively correlated with crown decline | (Aguayo et al., 2014) |
|  |  |  | Precipitation | No relation |  |
| 4 | *Phytophthora spp.* on Marri (*Corymbia calophylla*) | Australia | Temperature | Temperature increase was not related to canker incidence | (Paap et al., 2017) |
|  |  |  | Rainfall | Incidence showed slight increase with decreasing rainfall |  |
| 5 | *Dothistroma* needle blight caused by *Dothistroma septosporum* and *D. pini* in *Pinus spp.* | World | Temperature and precipitation | Historic and current reports show increase in the outbreak positively associated with increase in temperature and precipitation | (Woods et al., 2016) |
| 6 | Pine blister rust (*Cronartium ribicola*) on whitebark pine (*Pinus albicaulis*) | Western USA | Temperature and relative humidity | Prevalence of infection increased with high RH and increasing temperature until a certain threshold of 11^0^C and then sharply decreased | (Thoma et al., 2019) |
|  |  |  | Rainfall | Had no direct effect |  |
| **Disease, predicted** | | | | | |
| 1 | D. sapinea on Pinus spp. (2050-70) | Italy | Temperature | Outbreaks will increase in wider areas with a predicted increase in temperature | (Bosso et al., 2017) |
|  |  |  | Precipitation | Precipitation is among the drivers of outbreak in the wet areas, but no change was predicted |  |
| 2 | Pine Wilt Disease (Pinewood nematode, Bursaphelenchus xylophilus) (2026–2050) | Japan | Temperature and precipitation | Areas which will be highly affected by the disease will increase with predicted future precipitation and temperature | (Matsuhashi et al., 2020) |
| 3 | *D. pinea* on *Pinus spp*. (1990–2005 to 2030–2060) | France | Temperature | An increase in prevalence in 2030–2060 is predicted to be associated with an increase in daily minimum winter temperature | (Fabre et al., 2011) |
|  |  |  | Summer rain | A predicted decrease in summer rain decreases predicted prevalence, an overall increase in prevalence was predicted |  |
| 4 | Cedar leaf blight (Didymascella thujina) on western redcedar (*Thuja plicata*) (2020s to 2080s) | Canada | Rainfall | Predicted decrease in rainfall will decrease intensity is by the 2080s | (Gray et al., 2013) |
| **Insect pest, observed** | | | | | |
| 1 | Acute Oak Decline (Agrilus biguttatus) on Oak (*Quercus robur*) | England | Temperature and rainfall | An increase in temperature and decrease in rainfall were positively correlated with the area affected | (Brown et al., 2018) |
| 2 | Spruce budworm (*Choristoneura* spp.) on *Abies balsamea* and *Picea mariana* | North America | Temperature | Spruce budworm outbreaks were associated with warmer summer and cold spring,  Combined abiotic and biotic stress increased the severity of tree mortality | (De Grandpré et al., 2019) |
| 3 | *Tomicus piniperda* on Scots pine (*Pinus sylvestris*) | Spain | Climatic suitability model for both the pest and the host | Level of infestation is positively related to both the host and *T. piniperda* suitability | (Jaime et al., 2019) |
|  | *Ips sexdentatus* on Scots pine (*P. sylvestris*) |  |  | Infestation is negatively related to *I. sexdentatus* suitability |  |
| 4 | Pine caterpillars (*Dendrolimus spp.*) on *Pinus thunbergii, Pinus densiflora* and *Platycladus orientalis* forest | Shan dong, China | Drought (Standard precipitation index) | The area affected and intensity of outbreak decreased with increasing precipitation, drought significantly increased outbreak | (Bao et al., 2019) |
| 5 | Sirex woodwasp (*Sirex noctilio*) on *Pinus spp.* | Argentina | Drought | Affected trees and the resulting mortality increased with an increase in drought severity | (Lantschner et al., 2019) |
| 6 | Eurasian spruce bark beetle (*Ips typographus*) on Norway Spruce (*Picea abies*) | Eight European Countries | Rainfall and temperature | The volume of standing dead trees in relation to *I. typographus* infestation increased with a decrease in rainfall and an increase in summer temperature | (Marini et al., 2017) |
| 7 | Eurasian spruce bark beetle (*I. typographus*) on Norway Spruce (*P. abies*) | Slovakia | Temperature | Infestation rate increased with an increasing temperature up to a certain threshold and then decreased | (Mezei et al., 2017) |
| 8 | Mountain pine beetle (*Dendroctonus ponderosae*) on lodgepole (*Pinus contorta*) and whitebark pine (*P. albicaulis*) | Western USA | Temperature | Outbreaks occurred during relatively warmer years including relatively warmer winters which may improve winter survival of insects | (Creeden et al., 2014; Buotte et al., 2016; 2017) |
|  |  |  | Drought (Palmer Drought Severity Index, PDSI) | Multiyear drought preceded and continued during outbreaks and corresponded with increased tree mortality |  |
| 9 | Eurasian spruce bark beetle  (*I. typographus*) on Norway spruce (*P. abies*) | Austria | Drought (daily transpiration deficit, TDEF) and temperature | Probability of outbreak increased with drought and an increase in temperature. Stands in relatively wetter areas were more susceptible to beetle attack during drought than those in drier areas. | (Netherer et al., 2019) |
| 10 | Larch case­bearer (*Coleophora laricella*) and eastern larch beetle (*Dendroctonus simplex*) on eastern larch (*Larix laricina*) | Minnesota, USA | Temperature and precipitation | Larch case­bearer: outbreak increased with increasing minimum temperature and precipitation  Eastern larch beetle: outbreak and tree mortality increased with increases in minimum temperature, degree-day accumulation, and decrease in precipitation of previous years | (Ward and Aukema, 2019) |
| 11 | Western spruce budworm (*Choristoneura freeman*) on Douglas-fir (*Pseudotsuga menziesii*) | Western USA | Drought (PDSI and climatic water deficit, CWD) | Low PDSI and high CWD in the year of outbreak and years prior facilitated initiation in wetter areas  Outbreak initiation had no relation with drought in drier areas  Drought didn’t affect defoliation area growth rates after initiation in wetter areas  Increased precipitation had weak correlation with defoliation area increases in drier areas | (Xu et al., 2019) |
| 12 | Pine processionary moth (*Thaumetopoea pityocampa*) on *Pinus spp*. | Spain | Temperature | Winter minimum temperatures lower than -12^0^C decreased incidence of outbreak in the more susceptible *P. nigra* | (Gazol et al., 2019) |
| **Insect pest, predicted** | | | | | |
| 1 | Pine caterpillars (*Dendrolimus spp*.) on *P. thunbergii, P. densiflora* and *P. orientalis* forest | Shan dong, China | Precipitation | The area affected and intensity of outbreak will decrease with a predicted increase in precipitation | (Bao et al., 2019) |
| 2 | Mountain pine beetle (*D. ponderosae*) on whitebark pine (*P. albicaulis*) up to 2100 | Western USA | Temperature and precipitation | Despite regional variations, projections showing consistent increase in temperature and small decrease in summer precipitation tend to lead to an overall increasing climate suitability for mountain pine beetle outbreaks and the resulting white bark pine mortality | (Buotte et al., 2016; 2017) |
| 3 | Longhorned borer (*Phoracantha semipunctata)* on *Eucalyptus* | Australia | Drought | Outbreak will continue to increase with drought | (Seaton et al., 2015) |

* Reports which quantify damage mainly in terms of tree mortality and are summarized in Supplementary Table 3 are excluded from this table.

**This summary is limited to abiotic factors which are related to drought and heat stresses.

## Supplementary Table 2. Summary of landscape level tree mortality events reported in peer reviewed publications associated with drought, heat, pests, pathogens, and their combinations. *

| **S. No.** | **Study period** | **Continent** | **Country/ region** | **Main affected genera of trees** | **Biotic stress reported** | **Abiotic stress reported** | **Intensity of tree mortality** | **Reference** |
| --- | --- | --- | --- | --- | --- | --- | --- | --- |
| 1 | 2002 | Africa | Namibia, South Africa | *Aloe* | Not important | Hotter drought | Background | (Foden et al., 2007) |
| 2 | 1996-2014 | North America | Canada | *Abies* | Bark beetle | Temperature and moisture variation | Background | (Maclauchlan, 2016) |
| 3 | 2013 | Australia | Australia | *Eucalyptus* | Insect defoliator | Drought | Background | (Ross and Brack, 2015) |
| 4 | 1998 | Africa | South Africa | *Colophospermum* | Not reported | Drought | Catastrophic | (Macgregor and O'Connor, 2002) |
| 5 | 2014-2017 | Africa | South Africa | *Acacia, Combretum, Dichrostachys, Flueggea, Spirostachys, Terminalia, Euclea, Ehretia, Ziziphus, Commiphora, Grewia* | Not reported | Hotter Drought | Catastrophic | (Swemmer, 2020) |
| 6 | 2002-2005 | North America | USA | *Pinus, Juniperus* | Bark beetle | Hotter Drought | Catastrophic | (Floyd et al., 2009) |
| 7 | 2004–2016 | North America | USA | *Quercus, Fraxinus, Juniperus, Carya, Acer* | Pathogen | Hotter Drought | Catastrophic | (Gu et al., 2015; Agne et al., 2018) |
| 8 | 2009-2010 | Africa | South Africa | *Euphorbia* | Bark beetle, Pathogen | Temperature and moisture variation | Catastrophic | (Van Der Linde et al., 2012) |
| 9 | 2013-2014 | North America | USA | *Abies, Picea, Others* | Bark beetle, Pathogen | Temperature and moisture variation | Background | (Lalande et al., 2020) |
| 10 | 2004-2010 | North America | USA | *Fraxinus* | Wood borers | Not reported | Catastrophic | (Klooster et al., 2014) |
| 11 | 2012-2017 | Asia | Iran | *Quercus* | Wood borers, Pathogen | Hotter drought | Catastrophic | (Gheitury et al., 2020) |
| 12 | 2004-2011 | Asia | Japan | *Quercus, Fagus* | Wood borers, Pathogen | Not reported | Catastrophic | (Nakajima, 2019) |
| 13 | 2012-2016 | Europe | Czech Republic | *Picea* | Pathogen | Hotter drought | Catastrophic | (Holuša et al., 2018) |
| 14 | 2010-2016 | Europe | France, Belgium | *Fraxinus* | Pathogen | Not reported | Catastrophic | (Marcais et al., 2017) |
| 15 | 1999-2017 | Europe | Siberia | *Abies* | Bark beetle | Hotter Drought | Catastrophic | (Kharuk et al., 2019) |
| 16 | 2002-2016 | Europe | Italy | *Quercus* | Pathogen | Temperature and moisture variation | Catastrophic | (Colangelo et al., 2018) |
| 17 | 1999-2003 | North America | USA | *Pinus* | Bark beetle | Hotter drought | Catastrophic | (Breshears et al., 2005) |
| 18 | 2013 | Europe | Portugal | *Pinus* | Pathogen | Temperature and moisture variation | Catastrophic | (Calvão et al., 2019) |
| 19 | 2006-2018 | Asia | China | *Pinus* | Pathogen | Temperature and moisture variation | Catastrophic | (Gao et al., 2019) |
| 20 | 2017 | Asia | China | *Populus* | Pathogen | Drought, soil nutrients | Catastrophic | (Ji et al., 2019) |
| 21 | 2004-2010 | North America | USA | *Pinus* | Bark beetle | Hotter drought | Catastrophic | (Millar et al., 2012) |
| 22 | 2000-2008 | North America | USA | *Populus* | Bark beetle, wood borers, insect defoliators, Pathogen | Hotter drought | Catastrophic | (Worrall et al., 2008; Worrall et al., 2010) |
| 23 | 2007 | Europe | Spain | *Pinus* | Parasitic plant | Hotter drought | Catastrophic | (Galiano et al., 2010) |
| 24 | 2014-2015 | Asia | Japan | *Abies* | Bark beetle, Pathogen | Not reported | Catastrophic | (Takagi et al., 2018) |
| 25 | 2005-2015 | North America | USA | *Pinus, Juniperus* | Bark beetle, wood borer, insect defoliators, parasitic plant | Drought | Background | (Flake and Weisberg, 2019) |
| 26 | 2005-2011 | Australia | Australia | *Eucalyptus, Petalostigma, Melaleuca, Terminalia, Corymbia, Acacia, Others* | Not reported | Hotter drought | Catastrophic | (Fensham et al., 2015) |
| 27 | 2001-2005 | Europe | Sweden | *Pinus* | Bark beetle, Pathogen | Not reported | Catastrophic | (Sikström et al., 2011) |
| 28 | 2014-2017 | North America | USA | *Pinus, Abies, Calocedrus,* | Bark beetles | Hotter drought | Catastrophic | (Potter, 2017; Fettig et al., 2019) |
| 29 | 1970-2008 | North America | Canada | *Abies, Pinus, Populus, Picea, Others* | Insect defoliators | Temperature and moisture variation | Background | (Zhang et al., 2014a) |
| 30 | 1986-2006 | Asia | China | *Pinus, Quercus, Betula, Tilia, Others* | Not reported | Hotter drought | Background | (Zhang et al., 2014b) |
| 31 | 2011-2015 | North America | USA | *Pinus, Ulmus, Liquidambar, Quercus, Juniperus, Prosopis, Fraxinus, Celtis* | Bark beetle, Pathogen | Hotter drought | Catastrophic | (Moore et al., 2016; Klockow et al., 2018) |
| 32 | 1999-2006 | Europe | Russia | *Picea* | Bark beetle | Hotter Drought | Catastrophic | (Aakala et al., 2011) |
| 33 | 1998-2005 | North America | USA | *Pinus, Picea, Abies,*  *Pseudotsuga* | Not reported | Hotter drought | Background | (Oswald et al., 2016) |
| 34 | 1997-2007 | North America | USA | *Pinus, Pseudotsuga, Abies, Quercus, Others* | Bark beetle | Hotter drought | Catastrophic | (Gitlin et al., 2006; Floyd et al., 2009; Ganey and Vojta, 2011) |
| 35 | 1987-2006 | Europe | Spain | *Pinus, Quercus, Eucalyptus, Fagus, Castanea, Juniperus, Betula* | Insect defoliators, Pathogen | Hotter drought | Background | (Carnicer et al., 2011) |
| 36 | 1999 | Africa | Uganda | Many | Not reported | Drought | Catastrophic | (Lwanga, 2003) |
| 37 | 1998-1999 | South America | Argentina | *Nothofagus* | Bark beetle, Pathogen | Hotter drought | Catastrophic | (Suarez et al., 2004) |
| 38 | 1983-1999 | South America | Brazil | Many | Not reported | Drought | Background | (Williamson et al., 2000) |
| 39 | 1981-1990 | South America | Panama | Many | Not reported | Hotter drought | Background | (Condit et al., 1995) |
| 40 | 20 years | North America | USA | *Abies, Pinus, Pseudotsuga* | Bark beetle, wood borers, Pathogen, parasitic plant | Not reported | Background | (Filip and Goheen, 1982) |
| 41 | 1992-1995 | North America | Canada | *Abies, Tsuga, Thuja, Picea* | Insect defoliators | Not reported | Catastrophic | (Alfaro et al., 1999) |
| 42 | 2005 | North America | USA | *Quercus, Lithocarpus* | Pathogen | Not reported | Catastrophic | (Meentemeyer et al., 2008) |
| 43 | 1992-1996 | North America | USA | *Quercus, Liquidambar, Pinus* | Insect defoliators | Not reported | Background | (Eisenbies et al., 2007) |
| 44 | 1997-2001 | Europe | Sweden | *Pinus* | Insect defoliators and bark beetle | Not reported | Background | (Cedervind and Långström, 2003; Cedervind et al., 2003) |
| 45 | 2010 | North America | USA | *Tsuga* | Sap-sucking insects | Not reported | Background | (Kantola et al., 2014) |
| 46 | 2005-2015 | Europe | Latvia | *Fraxinus* | Pathogen | Not reported | Catastrophic | (Matisone et al., 2018) |
| 47 | 1977-1980 | Australia | New Zealand | *Nothofagus* | Woodborers, Insect defoliators | Drought | Catastrophic | (Hosking and Kershaw, 1985) |
| 48 | 1991-1996 | Australia | Australia | *Acacia, Casuarina, Corymbia, Eucalyptus, Lysiphyllum, others* | Not reported | Drought | Catastrophic | (Fensham and Holman, 1999) |
| 49 | 1997-1998 | Asia | Malaysia | *Many* | Not reported | Drought | Catastrophic | (Potts, 2003) |
| 50 | 2005 | South America | Amazonia countries | *Many* | Not reported | Hotter Drought | Background | (Phillips et al., 2009) |
| 51 | 2014 | Europe | Spain | *Pinus* | Bark beetle | Hotter Drought | Catastrophic | (De La Serrana et al., 2015) |
| 52 | 2012-2017 | North America | USA | *Quercus, Aesculus,*  *Fraxinus, others* | Not reported | Hotter Drought | Background | (Das et al., 2020) |
| 53 | 2000-2007 | North America | Canada | *Populus* | Wood borers and insect defoliators | Hotter Drought | Catastrophic | (Hogg et al., 2008; Michaelian et al., 2011) |
| 54 | 2002-2010 | North America | USA | *Populus* | Wood borers, pathogens | Hotter Drought | Catastrophic | (Ganey and Vojta, 2011; Zegler et al., 2012) |
| 55 | 2005-2006 | North America | Canada | *Populus, Abies, Picea* | Insect defoliators, 1980’s and 1990’s | Not reported | Catastrophic | (Man and Rice, 2010) |
| 56 | 1998-1999 | North America | USA | *Chamaecyparis* | Pathogen | Not reported | Background | (Jules et al., 2014) |

*We searched on google scholar using different combinations of the key words: “tree”, “forest”, “vegetation”, “plantation”, “massive”, “mortality”, “die-off”, “die-back” and “decline”. Reports from relevant reviews (Allen et al., 2010; 2015; McDowell et al., 2018) were also searched. Reports which do not indicate the spatial characteristics and intensity of mortality as well as those which include other exogenous mortality agents such as fire, flood, etc were excluded. In addition, seedling mortality, mortality due to experimental stress, long term natural mortality analyses were not included. Then, reports which clearly describe individual mortality event/events and can be approximated as landscape scale according to the classification given in Lugo and Scatena (1996) were selected. Reports describing different aspects of the same mortality event at the same location were considered as single reports.

## Supplementary Table 3. Examples of estimations of economic loss in forestry due to biotic and abiotic stresses

| **Country/region** | **Study period** | **Main trees** | **Biotic stress** | **Abiotic stress** | **Damage** | **Cost/loss category** | **Cost unit** | **Estimated cost/ loss** | **References** |
| --- | --- | --- | --- | --- | --- | --- | --- | --- | --- |
| USA | Annual | Many species | Insect Pests | Not reported | Observed tree mortality | Government expenditure | Lost revenue | $7,1 billion per year | (Aukema et al., 2011) |
| South Africa | 1923-1983 | Pine | *Sphaeropsis sapinea* | Hail | Observed tree mortality | Wood loss | Lost revenue Extrapolated from observed loss | ZAR 9.5 million per year | (Zwolinski et al., 1990) |
| Canada | 2010-2080 | Many species | Insect pest outbreaks | climate change scenarios | Predicted Tree mortality | Wood loss | Predicted loss and gain in GDP of different regions | $208.86 billion loss to $15.09 billion gain | (Ochuodho et al., 2012) |
| Italy, Spain, France, Portugal | 2008-2030 | Conifer trees | Pine wood nematode | Temperature | Predicted Tree mortality | Wood loss | Direct impact over 22 years | €22 to 27 billion | (Soliman et al., 2012) |
|  |  |  |  |  |  |  | Indirect impact (social welfare) | €218 to €369 million |  |
| New Zealand | Annual | Pine | *Dothistroma* needle blight | Not reported | Cost of control | Decrease in tree growth | Difference in loss between sprayed and unsprayed | $13.0 million  per year | (Watt et al., 2011) |
| USA | Sample based analysis | Pine | *Caliciopsis pinea,* | Not reported | Reduced quality of products | Downgraded timber quality | Average percent loss of revenue | 3.20% | (Costanza et al., 2019) |
| Italy | 1990-2001 | Many species | Pathogens and insect pests | Drought, frost, and others | Defoliation and growth decline | Impact on Ecosystem services | Economic loss per year | €1,04 Million per year | (Notaro et al., 2009) |
| USA | Historic and predicted | Pine | Bark beetles | Temperature | Benefit of basal area reduction treatment | Observed climate | Benefit of management under different scenarios | $7.75 to $95.69 per hectare | (Waring et al., 2009) |
|  |  |  |  |  |  | Predicted drought |  | $47.96 to $174.58 per hectare |  |

## Supplementary Table 4. Examples of key physiological and biochemical traits perturbed under combined biotic and abiotic stresses in forest trees

| **Response trait** | **Species of tree** | **Combination of stress** | **Change under individual stress** | | **Change under Combined Stress*** | **Reference** |
| --- | --- | --- | --- | --- | --- | --- |
|  |  |  | **Biotic** | **Abiotic** |  |  |
| Chlorophyll content | *Quercus brantii* | *Obolarina persica* and *Biscogniauxia mediteranea* and drought | Decrease | Decrease | Decrease | (Ghanbary et al., 2018) |
| Plant water potential | *Pinus sylvestris* | *Leptographium wingfieldii* and drought | No change | Decrease | Decrease | (Croisé et al., 2001) |
|  | *Eucalyptus globulus* | *Neofusicoccum eucalyptorum* and drought | Decrease | Decrease | Further decrease | (Barradas et al., 2018) |
|  | *Quercus brantii* | *Obolarina persica* and *Biscogniauxia mediteranea* and drought | Decrease | Decrease | Further decrease | (Ghanbary et al., 2017) |
|  | *Corymbia calophylla* | *Quambalaria coyrecup* and drought | Decrease | Decrease | Decrease | (Hossain et al., 2019) |
|  | *Quercus ilex* and *Quercus cerris* | *Phytophthora cinnamomi* and drought | No change | Decrease | Decrease | (Turco et al., 2004) |
| Stomatal conductance | *Pinus sylvestris* | *Leptographium wingfieldii* and drought | No change | Decrease | Decrease | (Croisé et al., 2001) |
|  | *Quercus brantii* | *Obolarina persica* and *Biscogniauxia mediteranea* and drought | Decrease | Decrease | Further decrease | (Ghanbary et al., 2017) |
|  | *Corymbia calophylla* | *Quambalaria coyrecup* and drought | Decrease | Decrease | Decrease | (Hossain et al., 2019) |
| Rate of photosynthesis | *Quercus brantii* | *Obolarina persica* and *Biscogniauxia mediteranea* and drought | Decrease | Decrease | Further decrease | (Ghanbary et al., 2017) |
|  | *Corymbia calophylla* | *Quambalaria coyrecup* and drought | Decrease | Decrease | Decrease | (Hossain et al., 2019) |
| Fv/Fm** | *Quercus brantii* | *Obolarina persica* and *Biscogniauxia mediteranea* and drought | Decrease | Decrease | Further decrease | (Ghanbary et al., 2017) |
| H_2_O_2_ | *Pinus nigra* | *Diplodia sapinea* and drought | Decrease | Increase | Decrease | (Sherwood et al., 2015) |
|  | *Quercus brantii* | *Obolarina persica* and *Biscogniauxia mediteranea* and drought | Increase | No change | Further increase | (Ghanbary et al., 2018) |
| Malondialdehyde | *Quercus brantii* | *Obolarina persica* and *Biscogniauxia mediteranea* and drought | Increase | Increase | Further increase | (Ghanbary et al., 2018) |
| Activity of antioxidant enzymes *** | *Quercus brantii* | *Obolarina persica* and *Biscogniauxia mediteranea* and drought | Increase | Increase | Increase | (Ghanbary et al., 2018) |
| Electrolyte leakage | *Quercus brantii* | *Obolarina persica* and *Biscogniauxia mediteranea* and drought | Increase | Increase | Further increase | (Ghanbary et al., 2018) |
| Chitinase activity | *Quercus brantii* | *Obolarina persica* and *Biscogniauxia mediteranea* and drought | Increase | No change | Further increase | (Ghanbary et al., 2018) |
| Proline | *Pinus nigra* | *Diplodia sapinea* and drought | Increase | Increase | Further increase | (Sherwood et al., 2015) |
|  | *Quercus brantii* | *Obolarina persica* and *Biscogniauxia mediteranea* and drought | Increase | Increase | Further increase | (Ghanbary et al., 2018) |
| Soluble sugars | *Eucalyptus globulus* | *Phoracantha semipunctata,* and drought | Not specified | Increase | Not specified | (Caldeira et al., 2002) |
|  | *Quercus brantii* | *Obolarina persica* and *Biscogniauxia mediteranea* and drought | Increase | Increase | Further increase | (Ghanbary et al., 2018) |
| Phenolics | *Corymbia calophylla* | *Quambalaria coyrecup* and drought (stem) | Increase | No change | Less increase | (Hossain et al., 2019) |
|  | *Pinus halepensis* | Pine weevil, (*Hylobius*  *abietis*) and drought | Decrease | No change | Less decrease | (Suárez-Vidal et al., 2019) |
|  | *Quercus brantii* | *Obolarina persica* and *Biscogniauxia mediteranea* and drought | Increase | Increase | Further increase | (Ghanbary et al., 2018) |
| Total terpene | *Corymbia calophylla* | *Quambalaria coyrecup* and drought (leaf) | Increase | No change | Less increase | (Hossain et al., 2019) |
|  | *Pinus halepensis* | Pine weevil (*Hylobius*  *abietis*) and drought (only diterpens) | Increase | No change | Increase (under moderate drought) | (Suárez-Vidal et al., 2019) |
|  | *Pinus contorta* and *Pinus banksiana* | *Grosmannia clavigera* and drought (only monoterpene) | Increase | No change | Increase | (Lusebrink et al., 2016) |

*Further increase/decrease refers to a further increase/reduction under combined stress, which is higher/lower in quantity than both of the individual stresses as differentiated from an increase/decrease similar in quantity to one or both of the individual stresses *i.e* the quantity in combined stress </>individual stresses </>control. Less increase/decrease refers to an increase/decrease, which is lower in quantity than either or both of the single stresses *i.e* the quantity in individual stresses </>combined stress </>control

** Fv/Fm= Maximum photochemical efficiency of photosystem II photochemistry

*** The antioxidant enzymes superoxide dismutase, peroxidases, and glutathione reductase with more or less the same trend as mentioned in the table

# References

Aakala, T., Kuuluvainen, T., Wallenius, T., and Kauhanen, H. (2011). Tree mortality episodes in the intact *Picea abies*‐dominated taiga in the Arkhangelsk region of northern European Russia. *Journal of Vegetation Science* 22(2)**,** 322-333.

Agne, M.C., Beedlow, P.A., Shaw, D.C., Woodruff, D.R., Lee, E.H., Cline, S.P., et al. (2018). Interactions of predominant insects and diseases with climate change in Douglas-fir forests of western Oregon and Washington, USA. *Forest Ecology and Management* 409**,** 317-332. doi: 10.1016/j.foreco.2017.11.004.

Aguayo, J., Elegbede, F., Husson, C., Saintonge, F.X., and Marçais, B. (2014). Modeling climate impact on an emerging disease, the *Phytophthora alni*‐induced alder decline. *Global change biology* 20(10)**,** 3209-3221.

Alfaro, R.I., Taylor, S., Brown, G., and Wegwitz, E. (1999). Tree mortality caused by the western hemlock looper in landscapes of central British Columbia. *Forest Ecology and Management* 124(2-3)**,** 285-291.

Allen, C.D., Breshears, D.D., and McDowell, N.G. (2015). On underestimation of global vulnerability to tree mortality and forest die‐off from hotter drought in the Anthropocene. *Ecosphere* 6(8)**,** 1-55.

Allen, C.D., Macalady, A.K., Chenchouni, H., Bachelet, D., McDowell, N., Vennetier, M., et al. (2010). A global overview of drought and heat-induced tree mortality reveals emerging climate change risks for forests. *Forest Ecology and Management* 259(4)**,** 660-684. doi: 10.1016/j.foreco.2009.09.001.

Aukema, J.E., Leung, B., Kovacs, K., Chivers, C., Britton, K.O., Englin, J., et al. (2011). Economic impacts of non-native forest insects in the continental United States. *PLoS one* 6(9).

Bao, Y., Wang, F., Tong, S., Na, L., Han, A., Zhang, J., et al. (2019). Effect of Drought on Outbreaks of Major Forest Pests, Pine Caterpillars (*Dendrolimus spp*.), in Shandong Province, China. *Forests* 10(3)**,** 264.

Barradas, C., Pinto, G., Correia, B., Castro, B., Phillips, A., and Alves, A. (2018). Drought× disease interaction in *Eucalyptus globulus* under *Neofusicoccum eucalyptorum* infection. *Plant Pathology* 67(1)**,** 87-96.

Bosso, L., Luchi, N., Maresi, G., Cristinzio, G., Smeraldo, S., and Russo, D. (2017). Predicting current and future disease outbreaks of *Diplodia sapinea* shoot blight in Italy: species distribution models as a tool for forest management planning. *Forest Ecology and Management* 400**,** 655-664.

Breshears, D.D., Cobb, N.S., Rich, P.M., Price, K.P., Allen, C.D., Balice, R.G., et al. (2005). Regional vegetation die-off in response to global-change-type drought. *Proceedings of the National Academy of Sciences* 102(42)**,** 15144-15148.

Brodde, L., Adamson, K., Julio Camarero, J., Castaño, C., Drenkhan, R., Lehtijärvi, A., et al. (2019). *Diplodia* tip blight on its way to the north: drivers of disease emergence in Northern Europe. *Frontiers in plant science* 9**,** 1818.

Brown, N., Vanguelova, E., Parnell, S., Broadmeadow, S., and Denman, S. (2018). Predisposition of forests to biotic disturbance: Predicting the distribution of Acute Oak Decline using environmental factors. *Forest Ecology and Management* 407**,** 145-154.

Buotte, P.C., Hicke, J.A., Preisler, H.K., Abatzoglou, J.T., Raffa, K.F., and Logan, J.A. (2016). Climate influences on whitebark pine mortality from mountain pine beetle in the Greater Yellowstone Ecosystem. *Ecological Applications* 26(8)**,** 2507-2524.

Buotte, P.C., Hicke, J.A., Preisler, H.K., Abatzoglou, J.T., Raffa, K.F., and Logan, J.A. (2017). Recent and future climate suitability for whitebark pine mortality from mountain pine beetles varies across the western US. *Forest Ecology and Management* 399**,** 132-142.

Caldeira, M.C., Fernandéz, V., Tomé, J., and Pereira, J.S. (2002). Positive effect of drought on longicorn borer larval survivl and growth on eucalyptus trunks. *Annals of Forest Science* 59(1)**,** 99-106.

Calvão, T., Duarte, C.M., and Pimentel, C.S. (2019). Climate and landscape patterns of pine forest decline after invasion by the pinewood nematode. *Forest ecology and management* 433**,** 43-51.

Carnicer, J., Coll, M., Ninyerola, M., Pons, X., Sanchez, G., and Penuelas, J. (2011). Widespread crown condition decline, food web disruption, and amplified tree mortality with increased climate change-type drought. *Proceedings of the National Academy of Sciences of the United States of America* 108(4)**,** 1474-1478. doi: 10.1073/pnas.1010070108.

Cedervind, J., and Långström, B. (2003). Tree mortality, foliage recovery and top-kill in stands of Scots pine (*Pinus sylvestris*) subsequent to defoliation by the pine looper (*Bupalus piniaria*). *Scandinavian journal of forest research* 18(6)**,** 505-513.

Cedervind, J., Pettersson, M., and Långström, B. (2003). Attack dynamics of the pine shoot beetle, *Tomicus piniperda* (Col.; Scolytinae) in Scots pine stands defoliated by *Bupalus piniaria* (Lep.; Geometridae). *Agricultural and forest entomology* 5(3)**,** 253-261.

Colangelo, M., Camarero, J.J., Borghetti, M., Gentilesca, T., Oliva, J., Redondo, M.-A., et al. (2018). Drought and *Phytophthora* are associated with the decline of oak species in southern Italy. *Frontiers in plant science* 9**,** 1595.

Condit, R., Hubbell, S.P., and Foster, R.B. (1995). Mortality rates of 205 neotropical tree and shrub species and the impact of a severe drought. *Ecological monographs* 65(4)**,** 419-439.

Costanza, K.K., Crandall, M.S., Rice, R.W., Livingston, W.H., Munck, I.A., and Lombard, K. (2019). Economic implications of a native tree disease, *Caliciopsis canker*, on the white pine (*Pinus strobus*) lumber industry in the northeastern United States. *Canadian Journal of Forest Research* 49(5)**,** 521-530.

Creeden, E.P., Hicke, J.A., and Buotte, P.C. (2014). Climate, weather, and recent mountain pine beetle outbreaks in the western United States. *Forest Ecology and Management* 312**,** 239-251.

Croisé, L., Lieutier, F., Cochard, H., and Dreyer, E. (2001). Effects of drought stress and high density stem inoculations with *Leptographium wingfieldii* on hydraulic properties of young Scots pine trees. *Tree Physiology* 21(7)**,** 427-436.

Das, A.J., Ampersee, N.J., Pfaff, A.H., Stephenson, N.L., Swiecki, T.J., Bernhardt, E.A., et al. (2020). Tree mortality in blue oak woodland during extreme drought in Sequoia National Park, California. *Madroño* 66(4)**,** 164-175.

De Grandpré, L., Kneeshaw, D.D., Perigon, S., Boucher, D., Marchand, M., Pureswaran, D., et al. (2019). Adverse climatic periods precede and amplify defoliator‐induced tree mortality in eastern boreal North America. *Journal of Ecology* 107(1)**,** 452-467.

De La Serrana, R.G., Vilagrosa, A., and Alloza, J. (2015). Pine mortality in southeast Spain after an extreme dry and warm year: interactions among drought stress, carbohydrates and bark beetle attack. *Trees* 29(6)**,** 1791-1804.

Eisenbies, M.H., Davidson, C., Johnson, J., Amateis, R., and Gottschalk, K. (2007). Tree mortality in mixed pine–hardwood stands defoliated by the European gypsy moth (*Lymantria dispar* L.). *Forest Science* 53(6)**,** 683-691.

Fabre, B., Piou, D., DESPREZ‐LOUSTAU, M.L., and Marcais, B. (2011). Can the emergence of pine *Diplodia* shoot blight in France be explained by changes in pathogen pressure linked to climate change? *Global Change Biology* 17(10)**,** 3218-3227.

Fensham, R., and Holman, J. (1999). Temporal and spatial patterns in drought-related tree dieback in Australian savanna. *Journal of Applied Ecology***,** 1035-1050.

Fensham, R.J., Fraser, J., MacDermott, H.J., and Firn, J. (2015). Dominant tree species are at risk from exaggerated drought under climate change. *Global change biology* 21(10)**,** 3777-3785.

Fettig, C.J., Mortenson, L.A., Bulaon, B.M., and Foulk, P.B. (2019). Tree mortality following drought in the central and southern Sierra Nevada, California, US. *Forest ecology and management* 432**,** 164-178.

Filip, G.M., and Goheen, D.J. (1982). Tree mortality caused by root pathogen complex in Deschutes National Forest, Oregon. *Plant Disease* 66(3)**,** 240-243.

Flake, S.W., and Weisberg, P.J. (2019). Fine‐scale stand structure mediates drought‐induced tree mortality in pinyon–juniper woodlands. *Ecological applications* 29(2)**,** e01831.

Floyd, M.L., Clifford, M., Cobb, N.S., Hanna, D., Delph, R., Ford, P., et al. (2009). Relationship of stand characteristics to drought‐induced mortality in three Southwestern piñon–juniper woodlands. *Ecological Applications* 19(5)**,** 1223-1230.

Foden, W., Midgley, G.F., Hughes, G., Bond, W.J., Thuiller, W., Hoffman, M.T., et al. (2007). A changing climate is eroding the geographical range of the Namib Desert tree Aloe through population declines and dispersal lags. *Diversity and Distributions* 13(5)**,** 645-653.

Galiano, L., Martínez-Vilalta, J., and Lloret, F. (2010). Drought-induced multifactor decline of Scots pine in the Pyrenees and potential vegetation change by the expansion of co-occurring oak species. *Ecosystems* 13(7)**,** 978-991.

Ganey, J.L., and Vojta, S.C. (2011). Tree mortality in drought-stressed mixed-conifer and ponderosa pine forests, Arizona, USA. *Forest Ecology and Management* 261(1)**,** 162-168.

Gao, R., Wang, Z., Wang, H., Hao, Y., and Shi, J. (2019). Relationship between Pine Wilt Disease Outbreaks and Climatic Variables in the Three Gorges Reservoir Region. *Forests* 10(9)**,** 816.

Gazol, A., Hernández-Alonso, R., and Camarero, J.J. (2019). Patterns and drivers of pine processionary moth defoliation in Mediterranean mountain forests. *Frontiers in Ecology and Evolution* 7**,** 458.

Ghanbary, E., Kouchaksaraei, M.T., Guidi, L., Mirabolfathy, M., Etemad, V., Sanavi, S.A.M.M., et al. (2018). Change in biochemical parameters of Persian oak (*Quercus brantii* Lindl.) seedlings inoculated by pathogens of charcoal disease under water deficit conditions. *Trees* 32(6)**,** 1595-1608.

Ghanbary, E., Tabari Kouchaksaraei, M., Mirabolfathy, M., Modarres Sanavi, S., and Rahaie, M. (2017). Growth and physiological responses of *Quercus brantii* seedlings inoculated with *Biscogniauxia mediterranea* and *Obolarina persica* under drought stress. *Forest Pathology* 47(5).

Gheitury, M., Heshmati, M., Noroozi, A., Ahmadi, M., and Parvizi, Y. (2020). Monitoring mortality in a semiarid forest under the influence of prolonged drought in Zagros region. *International Journal of Environmental Science and Technology***,** 1-12.

Gitlin, A.R., Sthultz, C.M., Bowker, M.A., Stumpf, S., Paxton, K.L., Kennedy, K., et al. (2006). Mortality gradients within and among dominant plant populations as barometers of ecosystem change during extreme drought. *Conservation Biology* 20(5)**,** 1477-1486.

Gray, L.K., Russell, J.H., Yanchuk, A.D., and Hawkins, B.J. (2013). Predicting the risk of cedar leaf blight (*Didymascella thujina*) in British Columbia under future climate change. *Agricultural and forest meteorology* 180**,** 152-163.

Gu, L., Pallardy, S.G., Hosman, K., and Sun, Y. (2015). Drought-influenced mortality of tree species with different predawn leaf water dynamics in a decade-long study of a central US forest.

Hogg, E., Brandt, J., and Michaelian, M. (2008). Impacts of a regional drought on the productivity, dieback, and biomass of western Canadian aspen forests. *Canadian Journal of Forest Research* 38(6)**,** 1373-1384.

Holuša, J., Lubojacký, J., Čurn, V., Tonka, T., Lukášová, K., and Horák, J. (2018). Combined effects of drought stress and *Armillaria* infection on tree mortality in Norway spruce plantations. *Forest ecology and management* 427**,** 434-445.

Hosking, G., and Kershaw, D.J. (1985). Red beech death in the Maruia Valley South Island, New Zealand. *New Zealand Journal of Botany* 23(2)**,** 201-211.

Hossain, M., Veneklaas, E.J., Hardy, G.E.S.J., and Poot, P. (2019). Tree host–pathogen interactions as influenced by drought timing: linking physiological performance, biochemical defence and disease severity. *Tree Physiology* 39(1)**,** 6-18.

Jaime, L., Batllori, E., Margalef-Marrase, J., Navarro, M.Á.P., and Lloret, F. (2019). Scots pine (*Pinus sylvestris* L.) mortality is explained by the climatic suitability of both host tree and bark beetle populations. *Forest Ecology and Management* 448**,** 119-129.

Ji, Y., Zhou, G., Li, Z., Wang, S., Zhou, H., and Song, X. (2019). Triggers of widespread dieback and mortality of poplar (*Populus spp*.) plantations across northern China. *Journal of Arid Environments***,** 104076.

Jules, E.S., Carroll, A.L., Garcia, A.M., Steenbock, C.M., and Kauffman, M.J. (2014). Host heterogeneity influences the impact of a non‐native disease invasion on populations of a foundation tree species. *Ecosphere* 5(9)**,** 1-17.

Kantola, T., Lyytikäinen-Saarenmaa, P., Coulson, R.N., Strauch, S., Tchakerian, M.D., Holopainen, M., et al. (2014). Spatial distribution of hemlock woolly adelgid induced hemlock mortality in the Southern Appalachians. *Open Journal of Forestry 4 (05)* 4(05)**,** 492-506.

Kharuk, V., Shushpanov, A., Petrov, I., Demidko, D., Im, S., and Knorre, A. (2019). Fir (*Abies sibirica* Ledeb.) Mortality in Mountain Forests of the Eastern Sayan Ridge, Siberia. *Contemporary Problems of Ecology* 12(4)**,** 299-309.

Klockow, P.A., Vogel, J.G., Edgar, C.B., and Moore, G.W. (2018). Lagged mortality among tree species four years after an exceptional drought in east Texas. *Ecosphere* 9(10)**,** e02455.

Klooster, W.S., Herms, D.A., Knight, K.S., Herms, C.P., McCullough, D.G., Smith, A., et al. (2014). Ash (*Fraxinus spp*.) mortality, regeneration, and seed bank dynamics in mixed hardwood forests following invasion by emerald ash borer (*Agrilus planipennis*). *Biological Invasions* 16(4)**,** 859-873.

Lalande, B.M., Hughes, K., Jacobi, W.R., Tinkham, W.T., Reich, R., and Stewart, J.E. (2020). Subalpine fir mortality in Colorado is associated with stand density, warming climates and interactions among fungal diseases and the western balsam bark beetle. *Forest Ecology and Management* 466**,** 118133.

Lantschner, M.V., Aukema, B.H., and Corley, J.C. (2019). Droughts drive outbreak dynamics of an invasive forest insect on an exotic host. *Forest ecology and management* 433**,** 762-770.

Lugo, A.E., and Scatena, F.N. (1996). Background and catastrophic tree mortality in tropical moist, wet, and rain forests. *Biotropica***,** 585-599.

Lusebrink, I., Erbilgin, N., and Evenden, M.L. (2016). The effect of water limitation on volatile emission, tree defense response, and brood success of *Dendroctonus ponderosae* in two pine hosts, lodgepole, and jack pine. *Frontiers in Ecology and Evolution* 4**,** 2.

Lwanga, J.S. (2003). Localized tree mortality following the drought of 1999 at Ngogo, Kibale National Park, Uganda. *African Journal of Ecology* 41(2)**,** 194-196.

Macgregor, S.D., and O'Connor, T.G. (2002). Patch dieback of *Colophospermum mopane* in a dysfunctional semi‐arid African savanna. *Austral Ecology* 27(4)**,** 385-395.

Maclauchlan, L. (2016). Quantification of *Dryocoetes confusus*-caused mortality in subalpine fir forests of southern British Columbia. *Forest Ecology and Management* 359**,** 210-220.

Man, R., and Rice, J.A. (2010). Response of aspen stands to forest tent caterpillar defoliation and subsequent overstory mortality in northeastern Ontario, Canada. *Forest Ecology and Management* 260(10)**,** 1853-1860.

Marcais, B., Husson, C., Cael, O., Dowkiw, A., Saintonge, F.-X., Delahaye, L., et al. (2017). Estimation of ash mortality induced by *Hymenoscyphus fraxineus* in France and Belgium. *Baltic Forestry* 23(1)**,** 159-167.

Marini, L., Økland, B., Jönsson, A.M., Bentz, B., Carroll, A., Forster, B., et al. (2017). Climate drivers of bark beetle outbreak dynamics in Norway spruce forests. *Ecography* 40(12)**,** 1426-1435.

Matisone, I., Matisons, R., Laiviņš, M., and Gaitnieks, T. (2018). Statistics of ash dieback in Latvia. *Silva Fennica* 52(1)**,** 6.

Matsuhashi, S., Hirata, A., Akiba, M., Nakamura, K., Oguro, M., Takano, K.T., et al. (2020). Developing a point process model for ecological risk assessment of pine wilt disease at multiple scales. *Forest Ecology and Management* 463**,** 118010.

McDowell, N., Allen, C.D., Anderson‐Teixeira, K., Brando, P., Brienen, R., Chambers, J., et al. (2018). Drivers and mechanisms of tree mortality in moist tropical forests. *New Phytologist* 219(3)**,** 851-869.

Meentemeyer, R., Rank, N., Shoemaker, D., Oneal, C., Wickland, A., Frangioso, K., et al. (2008). Impact of sudden oak death on tree mortality in the Big Sur ecoregion of California. *Biological invasions* 10(8)**,** 1243-1255.

Mezei, P., Jakuš, R., Pennerstorfer, J., Havašová, M., Škvarenina, J., Ferenčík, J., et al. (2017). Storms, temperature maxima and the Eurasian spruce bark beetle *Ips typographus*—An infernal trio in Norway spruce forests of the Central European High Tatra Mountains. *Agricultural and Forest Meteorology* 242**,** 85-95.

Michaelian, M., Hogg, E.H., Hall, R.J., and Arsenault, E. (2011). Massive mortality of aspen following severe drought along the southern edge of the Canadian boreal forest. *Global Change Biology* 17(6)**,** 2084-2094.

Millar, C.I., Westfall, R.D., Delany, D.L., Bokach, M.J., Flint, A.L., and Flint, L.E. (2012). Forest mortality in high-elevation whitebark pine (*Pinus albicaulis*) forests of eastern California, USA; influence of environmental context, bark beetles, climatic water deficit, and warming. *Canadian Journal of Forest Research* 42(4)**,** 749-765.

Moore, G.W., Edgar, C.B., Vogel, J.G., Washington‐Allen, R.A., March, R.G., and Zehnder, R. (2016). Tree mortality from an exceptional drought spanning mesic to semiarid ecoregions. *Ecological Applications* 26(2)**,** 602-611.

Nakajima, H. (2019). Region-wide mass mortality of Japanese oak due to ambrosia beetle infestation: Mortality factors and change in oak abundance. *Forest ecology and management* 449**,** 117468.

Netherer, S., Panassiti, B., Pennerstorfer, J., and Matthews, B. (2019). Acute drought is an important driver of bark beetle infestation in Austrian Norway spruce stands. *Frontiers in Forests and Global Change* 2**,** 39.

Notaro, S., Paletto, A., and Raffaelli, R. (2009). Economic impact of forest damage in an Alpine environment. *Acta Silvatica et Lignaria Hungarica: An International Journal in Forest, Wood and Environmental Sciences* 5**,** 131-143.

Ochuodho, T.O., Lantz, V.A., Lloyd-Smith, P., and Benitez, P. (2012). Regional economic impacts of climate change and adaptation in Canadian forests: A CGE modeling analysis. *Forest Policy and Economics* 25**,** 100-112. doi: 10.1016/j.forpol.2012.08.007.

Oswald, B., Dugan, S., Balice, R., and Unger, D. (2016). Overstory Tree Mortality in Ponderosa Pine and Spruce-Fir Ecosystems Following a Drought in Northern New Mexico. *Forests* 7(10)**,** 225.

Paap, T., Brouwers, N.C., Burgess, T.I., and Hardy, G.E.S.J. (2017). Importance of climate, anthropogenic disturbance and pathogens (*Quambalaria coyrecup* and *Phytophthora spp*.) on marri (*Corymbia calophylla*) tree health in southwest Western Australia. *Annals of Forest Science* 74(3)**,** 62.

Phillips, O.L., Aragão, L.E., Lewis, S.L., Fisher, J.B., Lloyd, J., López-González, G., et al. (2009). Drought sensitivity of the Amazon rainforest. *Science* 323(5919)**,** 1344-1347.

Potter, C.S. (2017). Satellite image mapping of tree mortality in the Sierra Nevada region of California from 2013 to 2016.

Potts, M.D. (2003). Drought in a Bornean everwet rain forest. *Journal of Ecology* 91(3)**,** 467-474.

Ross, C., and Brack, C. (2015). *Eucalyptus viminalis* dieback in the Monaro region, NSW. *Australian Forestry* 78(4)**,** 243-253.

Seaton, S., Matusick, G., Ruthrof, K.X., and Hardy, G.E.S.J. (2015). Outbreak of *Phoracantha semipunctata* in response to severe drought in a Mediterranean *Eucalyptus* forest. *Forests* 6(11)**,** 3868-3881.

Sherwood, P., Villari, C., Capretti, P., and Bonello, P. (2015). Mechanisms of induced susceptibility to *Diplodia* tip blight in drought-stressed Austrian pine. *Tree physiology* 35(5)**,** 549-562.

Sikström, U., Jacobson, S., Pettersson, F., and Weslien, J. (2011). Crown transparency, tree mortality and stem growth of *Pinus sylvestris*, and colonization of *Tomicus piniperda* after an outbreak of *Gremmeniella abietina*. *Forest ecology and management* 262(12)**,** 2108-2119.

Soliman, T., Mourits, M.C., Van Der Werf, W., Hengeveld, G.M., Robinet, C., and Lansink, A.G.O. (2012). Framework for modelling economic impacts of invasive species, applied to pine wood nematode in Europe. *PLoS One* 7(9).

Suárez-Vidal, E., Sampedro, L., Voltas, J., Serrano, L., Notivol, E., and Zas, R. (2019). Drought stress modifies early effective resistance and induced chemical defences of Aleppo pine against a chewing insect herbivore. *Environmental and Experimental Botany* 162**,** 550-559.

Suarez, M.L., Ghermandi, L., and Kitzberger, T. (2004). Factors predisposing episodic drought‐induced tree mortality in Nothofagus–site, climatic sensitivity and growth trends. *Journal of Ecology* 92(6)**,** 954-966.

Swemmer, A. (2020). Locally high, but regionally low: the impact of the 2014–2016 drought on the trees of semi-arid savannas, South Africa. *African Journal of Range & Forage Science* 37(1)**,** 31-42.

Takagi, E., Masaki, D., Kanai, R., Sato, M., and Iguchi, K. (2018). Mass mortality of *Abies veitchii* caused by *Polygraphus proximus* associated with tree trunk diameter in Japan. *Forest ecology and management* 428**,** 14-19.

Thoma, D.P., Shanahan, E.K., and Irvine, K.M. (2019). Climatic Correlates of White Pine Blister Rust Infection in Whitebark Pine in the Greater Yellowstone Ecosystem. *Forests* 10(8)**,** 666.

Turco, E., Close, T., Fenton, R., and Ragazzi, A. (2004). Synthesis of dehydrin-like proteins in Quercus ilex L. and Quercus cerris L. seedlings subjected to water stress and infection with Phytophthora cinnamomi. *Physiological and molecular plant pathology* 65(3)**,** 137-144.

Van Der Linde, J.A., Roux, J., Wingfield, M.J., and Six, D.L. (2012). Die-off of giant *Euphorbia* trees in South Africa: symptoms and relationships to climate. *South African journal of botany* 83**,** 172-185.

Ward, S.F., and Aukema, B.H. (2019). Anomalous outbreaks of an invasive defoliator and native bark beetle facilitated by warm temperatures, changes in precipitation and interspecific interactions. *Ecography* 42(5)**,** 1068-1078.

Waring, K.M., Reboletti, D.M., Mork, L.A., Huang, C.-H., Hofstetter, R.W., Garcia, A.M., et al. (2009). Modeling the impacts of two bark beetle species under a warming climate in the southwestern USA: ecological and economic consequences. *Environmental management* 44(4)**,** 824-835.

Watt, M., Bulman, L., and Palmer, D. (2011). The economic cost of *Dothistroma* needle blight to the New Zealand forest industry. *New Zealand Journal of Forestry* 56(1)**,** 20-22.

Williamson, G.B., Laurance, W.F., Oliveira, A.A., Delamônica, P., Gascon, C., Lovejoy, T.E., et al. (2000). Amazonian tree mortality during the 1997 El Nino drought. *Conservation Biology* 14(5)**,** 1538-1542.

Woods, A., Martín‐García, J., Bulman, L., Vasconcelos, M.W., Boberg, J., La Porta, N., et al. (2016). *Dothistroma* needle blight, weather and possible climatic triggers for the disease's recent emergence. *Forest Pathology* 46(5)**,** 443-452.

Worrall, J.J., Egeland, L., Eager, T., Mask, R.A., Johnson, E.W., Kemp, P.A., et al. (2008). Rapid mortality of *Populus tremuloides* in southwestern Colorado, USA. *Forest Ecology and Management* 255(3-4)**,** 686-696.

Worrall, J.J., Marchetti, S.B., Egeland, L., Mask, R.A., Eager, T., and Howell, B. (2010). Effects and etiology of sudden aspen decline in southwestern Colorado, USA. *Forest Ecology and Management* 260(5)**,** 638-648.

Xu, B., Hicke, J.A., and Abatzoglou, J.T. (2019). Drought and Moisture Availability and Recent Western Spruce Budworm Outbreaks in the Western United States. *Forests* 10(4)**,** 354.

Zegler, T.J., Moore, M.M., Fairweather, M.L., Ireland, K.B., and Fulé, P.Z. (2012). *Populus tremuloides* mortality near the southwestern edge of its range. *Forest Ecology and Management* 282**,** 196-207.

Zhang, X., Lei, Y., Ma, Z., Kneeshaw, D., and Peng, C. (2014a). Insect‐induced tree mortality of boreal forests in eastern Canada under a changing climate. *Ecology and evolution* 4(12)**,** 2384-2394.

Zhang, X., Lei, Y., Pang, Y., Liu, X., and Wang, J. (2014b). Tree mortality in response to climate change induced drought across Beijing, China. *Climatic change* 124(1-2)**,** 179-190.

Zwolinski, J., Swart, W., and Wingfield, M. (1990). Economic impact of a post‐hail outbreak of dieback induced by *Sphaeropsis sapinea*. *European Journal of Forest Pathology* 20(6‐7)**,** 405-411.
